# Non-invasive detection of severe neutropenia in chemotherapy patients by optical imaging of nailfold microcirculation

**DOI:** 10.1038/s41598-018-23591-0

**Published:** 2018-03-28

**Authors:** Aurélien Bourquard, Alberto Pablo-Trinidad, Ian Butterworth, Álvaro Sánchez-Ferro, Carolina Cerrato, Karem Humala, Marta Fabra Urdiola, Candice Del Rio, Betsy Valles, Jason M. Tucker-Schwartz, Elizabeth S. Lee, Benjamin J. Vakoc, Timothy P. Padera, María J. Ledesma-Carbayo, Yi-Bin Chen, Ephraim P. Hochberg, Martha L. Gray, Carlos Castro-González

**Affiliations:** 1 Research Laboratory of Electronics, Massachusetts Institute of Technology, Cambridge, MA USA; 2 Biomedical Image Technologies, Universidad Politécnica de Madrid and CIBER-BBN, Madrid, Spain; 3 Centro Integral en Neurociencias HM CINAC, HM Hospitales, Móstoles, Madrid, Spain; 4 Departamento de Hematología, Hospital Universitario La Paz, Madrid, Spain; 5 Massachusetts General Hospital Cancer Center, Harvard Medical School, Boston, MA USA; 6 Institute of Medical Engineering and Science, Massachusetts Institute of Technology, Cambridge, MA USA; 7 Wellman Center for Photomedicine, Harvard Medical School and Massachusetts General Hospital, Boston, MA USA; 8 Edwin L. Steele Laboratories, Department of Radiation Oncology, Massachusetts General Hospital and Harvard Medical School, Boston, MA USA

## Abstract

White-blood-cell (WBC) assessment is employed for innumerable clinical procedures as one indicator of immune status. Currently, WBC determinations are obtained by clinical laboratory analysis of whole blood samples. Both the extraction of blood and its analysis limit the accessibility and frequency of the measurement. In this study, we demonstrate the feasibility of a non-invasive device to perform point-of-care WBC analysis without the need for blood draws, focusing on a chemotherapy setting where patients’ neutrophils—the most common type of WBC—become very low. In particular, we built a portable optical prototype, and used it to collect 22 microcirculatory-video datasets from 11 chemotherapy patients. Based on these videos, we identified moving optical absorption gaps in the flow of red cells, using them as proxies to WBC movement through nailfold capillaries. We then showed that counting these gaps allows discriminating cases of severe neutropenia (<500 neutrophils per µL), associated with increased risks of life-threatening infections, from non-neutropenic cases (>1,500 neutrophils per µL). This result suggests that the integration of optical imaging, consumer electronics, and data analysis can make non-invasive screening for severe neutropenia accessible to patients. More generally, this work provides a first step towards a long-term objective of non-invasive WBC counting.

## Introduction

White-blood-cell (WBC) status is used as one indicator of immunological status in the diagnosis and treatment of multiple medical conditions, including cancer^1^, infectious diseases^2,3^, sepsis^4^, autoimmune disorders^5^, and in the use of immunosuppressant drugs^6^. However, all current methods require a blood sample which involves a visit to a healthcare center and trained clinical personnel, even with finger-prick technologies^7,8^. This limitation inherently restricts how frequently and quickly monitoring can be performed. In addition, traditional blood testing requires specific reagents and sterile conditions, which precludes applicability in resource-limited environments^9^. By contrast, a non-invasive approach to WBC measurement could circumvent these requirements, and may enable quick and self-administered testing in outpatient care, including at the patient’s home or walk-in clinics, drawing parallels to existing non-invasive technologies for the monitoring of blood oxygen saturation levels^10^.

As a first step towards non-invasive WBC analysis, we focused on whether one could non-invasively screen for severe neutropenia^11^, defined as low levels of neutrophils (<500 per µL)—the most common WBC type. This condition is one of the main toxicities in patients receiving common chemotherapy regimens. It is responsible for a significant amount of morbidity and a significant risk of mortality because of its associated increased risk of infection^12^. Yet, the monitoring of severe neutropenia is currently insufficient for the aforementioned reasons. This barrier to rapid clinical care interferes with the timely life-saving interventions of prophylactic antibiotics or granulocyte-colony stimulating factors in afebrile patients with prolonged severe neutropenia. In that regard, a non-invasive method could substantially impact the outpatient care and management of patients at high risk for severe neutropenia-related immunosuppression^13,14^.

Our approach aims to provide a screening tool for severe neutropenia based on non-invasive and portable optical visualization of capillaries. In doing so, we do not intend to be as accurate as current laboratory tests or replace the way clinical decisions are ultimately made. Rather, our approach could be employed as a trigger where patients would be referred to state-of-the-art blood tests if severe neutropenia is detected. Conceptually, when capillary diameter approaches WBC diameter (10–20 um), the latter completely fills the capillary lumen. This causes a red-blood-cell (RBC) depletion downstream of the WBC in the microcirculation where WBCs flow slower than RBCs^15^. Therefore, if the illumination renders WBCs transparent and RBCs dark—as it occurs at specific wavelengths—the passage of a WBC will appear as an optical absorption gap in the continuous RBC stream that moves through the capillary.

Using white-light transillumination microscopy, Schmid-Schoenbein *et al*. observed this “gap” phenomenon in a rabbit ear window model. They showed that RBCs accumulated upstream of WBCs with a RBC-depleted area downstream^15^ when the capillary and WBC diameters were comparable. Sinclair *et al*.^16^ observed the same phenomenon in rat-cremaster and bat-wing microcirculation, using blue-light transillumination to maximize contrast between RBCs—peak absorption for oxy- and deoxyhemoglobin is 420-nm blue^17^—and low-absorption regions lacking RBCs. By observing the flow of RBCs over time, they first identified the capillary morphology, and then brighter regions associated with optical-absorption gaps inside the capillary lumen. They also used fluorescent labeling to confirm that gaps were associated with WBCs.

The idea that such absorption gaps relate to WBCs was investigated in humans by taking advantage of the *blue entoptic phenomenon* where WBCs let blue light through as they flow in front of the retina, thus creating bright spots that the subject can see^18^. In other previous studies^19–21^, subjects had their eyes illuminated with blue light and were asked how many bright spots they perceived. Group differences in amounts of perceived spots between baseline, leukopenic, and leukocytotic subjects—related to normal, abnormally low, and abnormally high ranges of WBC counts—were reported. A limitation of these methods, however, is their reliance on subject self-assessment, which is prone to individual biases and poor repeatability. This does not make them amenable for clinical screening.

Overall, these findings suggest that flowing gaps in capillaries could provide a basis for a new method to assess WBCs non-invasively. Our implementation focused on nailfold capillaries that are superficial, *i*.*e*., 50–100-µm deep^22^, run parallel to the skin surface, so that they can be visualized non-invasively with simple, affordable optical equipment^23–27^, and have diameters comparable to WBC size^27^. Similar to existing non-invasive devices that measure features of blood, our method relied on the light-absorption properties of hemoglobin. However, in contrast to our approach, existing devices do not obtain any spatially resolved information (*i*.*e*., images), which we require in order to see flowing gaps. For instance, CO-oximetry^28^ and occlusion spectroscopy^29,30^ measure the bulk attenuation of light transmitted at a number of wavelengths through the finger wherein differences in oxygenation of hemoglobin lead to spectral differences that can be resolved to a measurement. In addition, whereas hemoglobin devices use red and infrared wavelengths (to ensure transmission through the finger, while being sensitive to oxygenated versus deoxygenated hemoglobin), we used wavelengths in the deep-blue range (420 nm) to maximize the contrast between the red blood cells and flow gaps in the superficial capillaries of interest.

**Figure 1:**
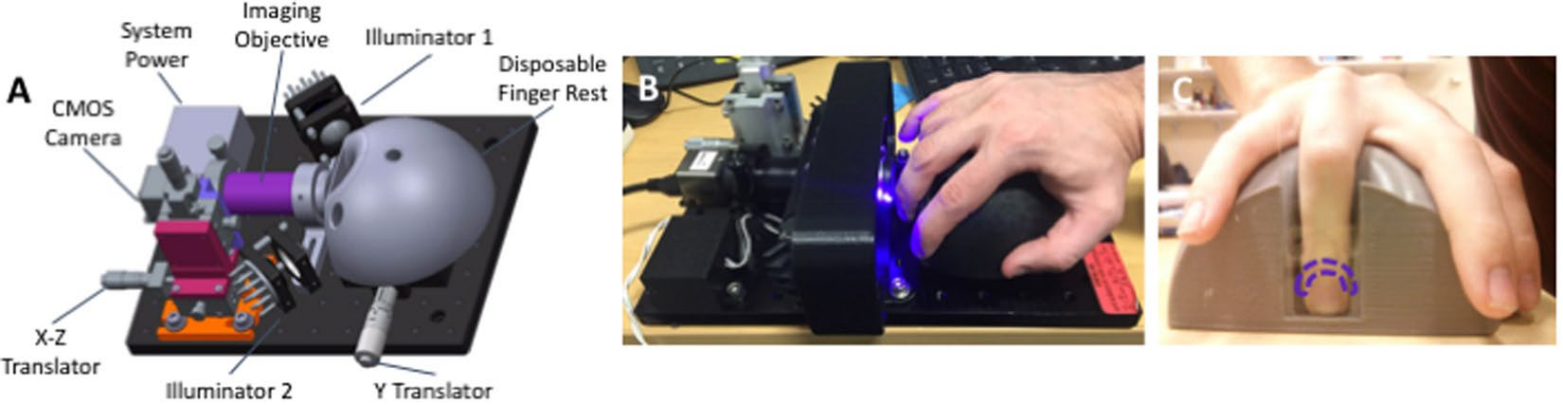
Clinical prototype. (**a**) 3D model of the custom-made portable prototype employed to record microscopy videos of the microcirculation in nailfold capillaries of patients, with its different components. (**b**) Patients place their ring finger in a 3D-printed holder, which plays a dual role: achieving stability throughout the one-minute recording duration and holding the oil employed for optical coupling. (**c**) The finger is placed so that illumination and imaging is directed at the nailfold area (dashed purple line).

**Figure 2:**
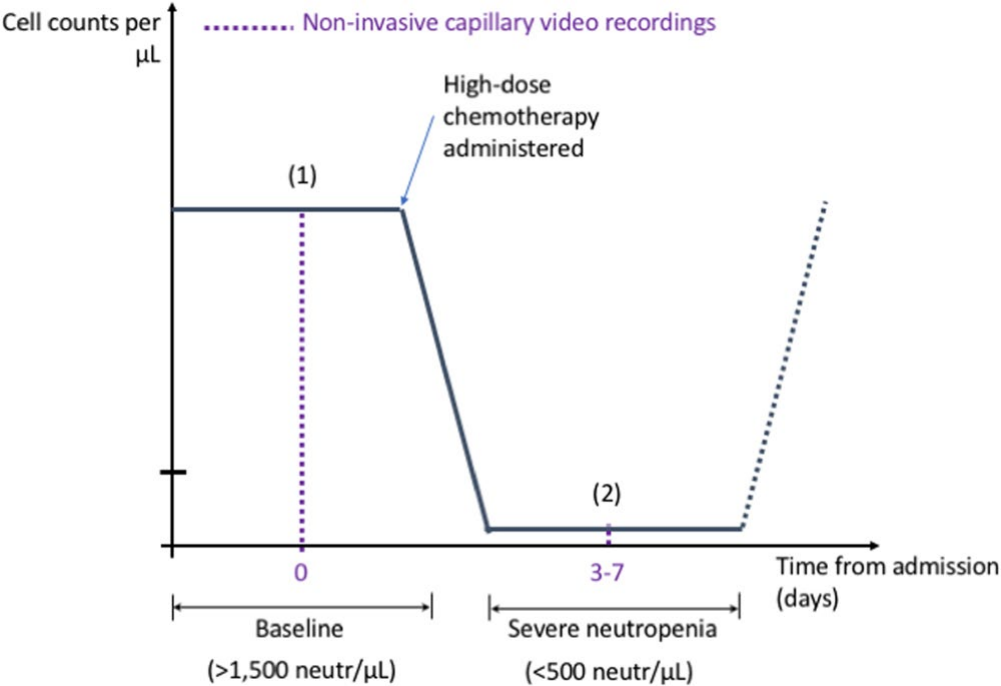
Patient acquisition time points. The patients enrolled in our study are undergoing an ASCT, process that results in a predictable evolution of their neutrophil counts due to the controlled administration of chemotherapy. This provides an opportunity to record capillary videos at two different time points for each patient: (1) baseline (>1,500 neutrophils/µL) and (2) severe neutropenia (<500 neutrophils/µL).

**Figure 3:**
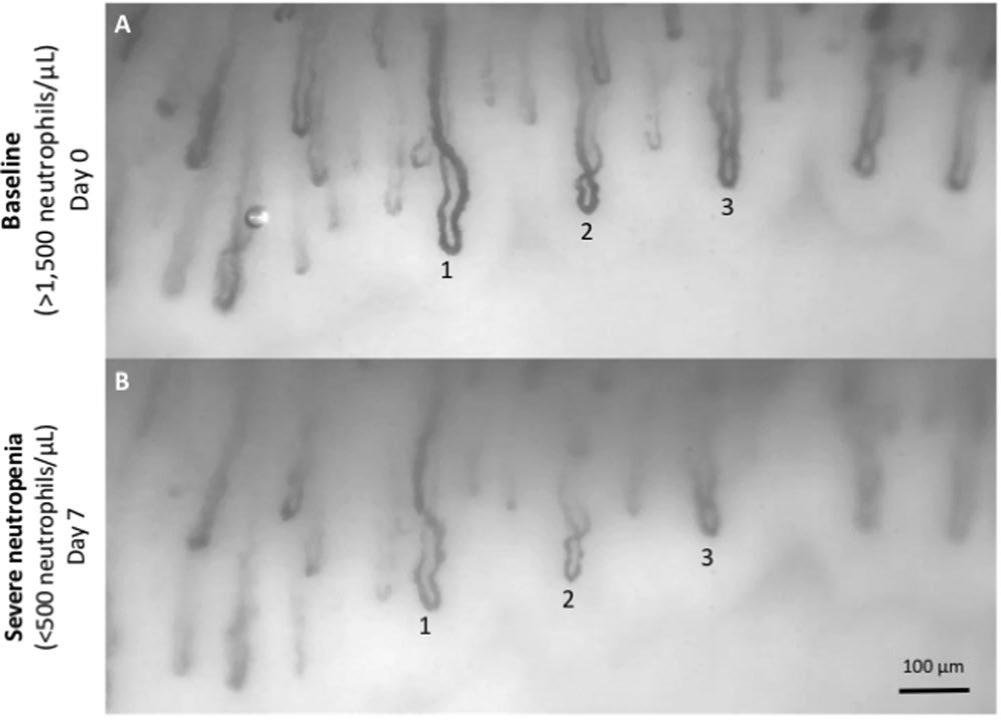
Example of raw acquisitions. This pair of wide-field videos (Supplementary Movie S1) was acquired with our optical prototype from one ASCT patient at two time points where the same capillaries can be observed (three numbered capillary pairs shown). (**a**) Baseline (neutrophils > 1,500/µL). (**b**) Severe neutropenia (neutrophils < 500/µL).

**Figure 4:**
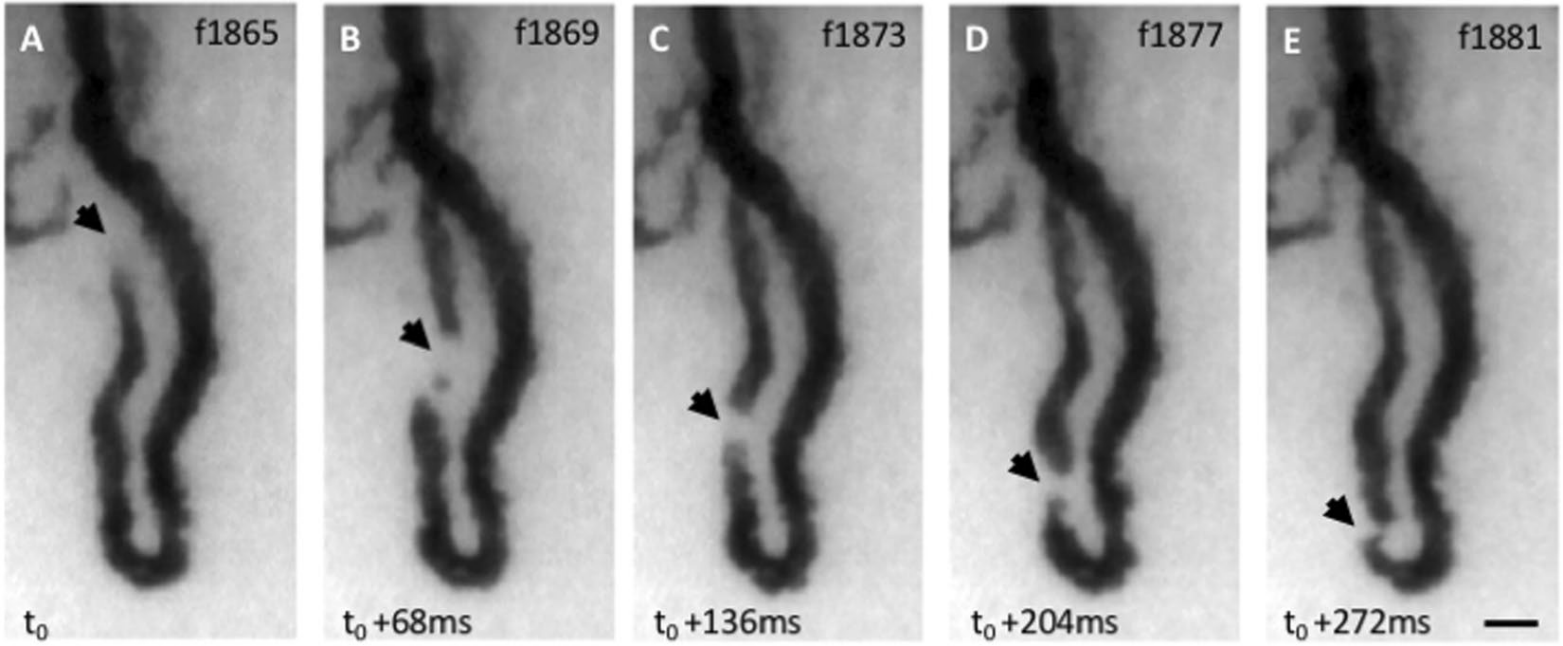
Example of event. (**a**–**e**) The image sequence shows several raw frames of a video (Supplementary Movie S2) centered on one capillary acquired with our prototype on one of the patients at baseline. The dark loop corresponds to the capillary vessel filled with RBCs that absorbs light at the illumination wavelength. An optical absorption gap in the microcirculation, approximately the same size as the capillary width (~15 µm) can be observed flowing through the arterial limb of the capillary (black triangular arrowheads). Frame numbers are labeled at the top right corner. The frame rate was 60 FPS and the exposure time 16.7 ms. The contrast was adjusted for the ROI shown.

**Figure 5:**
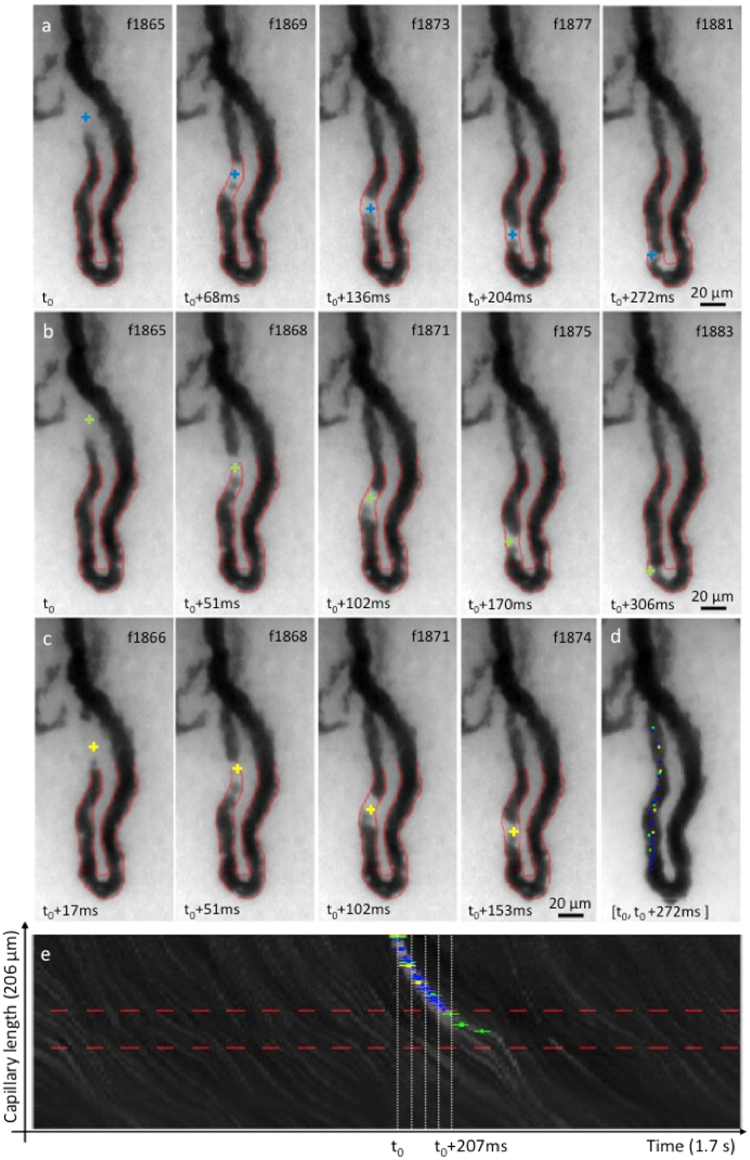
Blind event rating. Three raters independently labeled one same event they observed in one of the 106 capillaries of the study. (**a**–**c**) Capillary-video frames (indexed top right) with cross-shaped event marks from rater 1 (blue), 2 (green) and 3 (yellow). (**d**) Aggregated positions of all event marks from all three raters. (**e**) ST map displaying the recorded brightness levels along the segmented capillary length (vertical axis) as a function of time (horizontal axis) for a 1.7-second interval around the event of interest. A bright trajectory created by the passage of the event is clearly identified in the center of the ST map. Blue, green, and yellow crosses correspond to the ST coordinates where each rater labeled the event.

**Figure 6:**
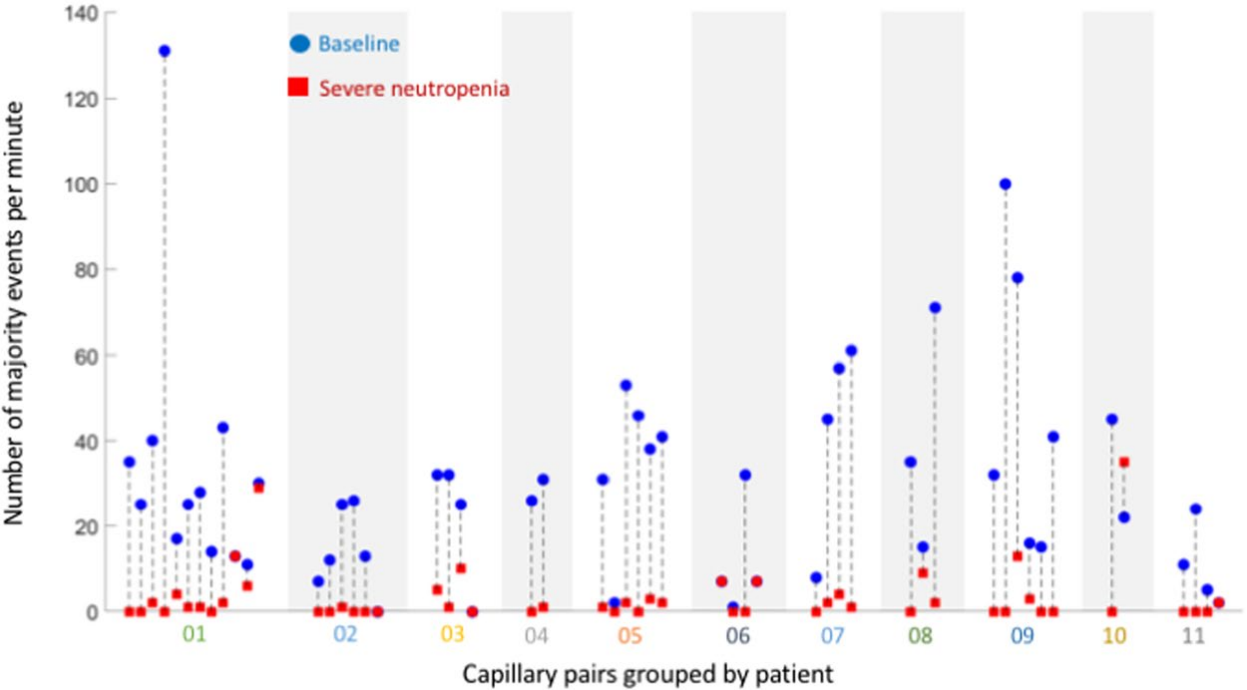
Number of majority events per minute in all studied capillary pairs. Capillary counts at baseline (blue dots) showed a statistically significant difference compared to the corresponding values during severe neutropenia (red squares). All capillaries were analyzed at both timepoints (53 pairs in total; black dotted lines). Only majority events were considered to maximize the objectivity of the event selection.

**Figure 7:**
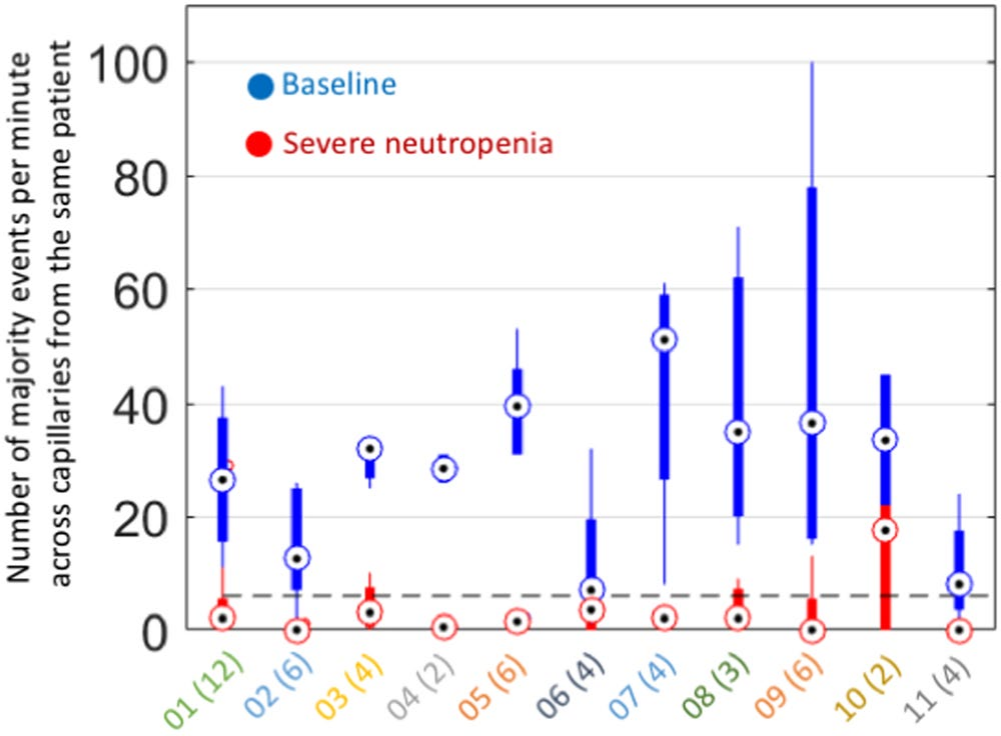
Discrimination between baseline and severe neutropenia. The median numbers of majority events observed per minute, when averaging across all available capillaries per patient, allow discriminating between baseline (blue dots) and severe neutropenia (red dots) for the 22 video datasets and 11 patients of our study. The corresponding cross-capillary variability is also shown for each patient (blue and red bars with notch extremes determined as *q*_*2*_ ± 1.57(*q*_3_ − *q*_1_)/*N*^1/2^, where the *q*_*i*_ are the respective quartiles, and where *N* = 11 is the amount of paired data points). The optimal threshold to separate baseline from severe neutropenia was seven events per capillary minute (dotted black line). The X-axis is labeled with the patient IDs together with their amount of analyzed capillaries in brackets. The median amount of capillaries used per patient was four. The difference in patient counts between baseline and severe neutropenia was statistically significant.

In this work, we demonstrate the ability of optical imaging to screen for severe neutropenia in human subjects by counting *events* defined as instances of moving optical absorption gaps in the nailfold microcirculation. To do so, we devised a portable prototype to produce optical microscopy videos of capillaries (Fig. 1). Our design maximizes RBC-to-non-RBC contrast over multiple capillaries within one field of view (FOV) while ensuring adequate resolution, depth of focus, stability, and frame rate. Based on this prototype, we conducted a clinical study (Fig. 2) involving 11 patients undergoing high-dose chemotherapy and autologous stem-cell transplant (ASCT)^31^. For each patient, we acquired one-minute videos of the same capillaries at two time points: pre-chemotherapy baseline (>1,500 neutrophils per µL) and severe neutropenia (<500 neutrophils per µL) (Fig. 3). Based on these data, we developed and validated a method to tag events (Figs 4 and 5) and count them to discriminate between baseline and severe neutropenia across patients (Figs 6–7).

## Methods

### Study design

We conducted a pilot diagnostic validation study to test the a-priori hypothesis that our approach allows the classification of severe neutropenia (<500 neutrophils/µL) versus the baseline status (>1,500 neutrophils/µL) in patients. We enrolled a cohort of patients undergoing high-dose chemotherapy followed by ASCT. The kinetics of neutrophil values in these patients was predictable because the intensity of the chemotherapy applied prior to transplantation ensures the passage through a severe-neutropenia stage followed by recovery (Fig. 2). In this proof-of-concept study, no power analysis was carried out and a convenience sample of 22 measurements from 11 subjects, as selected from our initial patient pool (see “Data collection”), was considered sufficient to test the study hypothesis. Non-parametric tests were used as well as receiver-operating-characteristic curve analyses. All human raters who analyzed the data were blinded (see “Capillary selection” and “Event rating”).

In total, 26 patients were recruited, with 17 and 9 patients from the Massachusetts General Hospital (MGH), Boston, MA, USA, and Hospital Universitario La Paz (HULP), Madrid, Spain, respectively. Each recruited patient signed an informed consent. All the information obtained was anonymous and the participants were not identifiable.

The patient inclusion criteria used for recruitment were the following: (a) patients must have a scheduled ASCT of hematopoietic progenitors; (b) patients must be 18 years or older; (c) patients must have the ability to understand and the willingness to sign a written consent document; (d) at baseline, patients must have a leukocyte count equal to or greater than 3,000 cells/µL and a neutrophil count equal to or greater than 1,500 cells/µL. Patients were excluded if suffering from myelodysplasia or from a history of allergic reactions to components of chemical compositions similar to the oil used for optical coupling in our clinical device, or if their skin phototype was larger than four on the Fitzpatrick scale^32^.

The MGH clinical study was approved by the Dana-Farber/Harvard Cancer Center institutional review board, and by the MIT Committee on the Use of Humans as Experimental Subjects as Protocol #1610717680. This study was also registered and posted at clinicaltrials.gov (NCT02512666) starting from July 31, 2015. The HULP clinical study was approved by the HULP Ethics Committee in the document HULP PI-2353. The analysis of anonymized data from these pilot studies was also approved by the Ethics Committee of Universidad Politécnica de Madrid and Massachusetts Institute of Technology. All experiments were performed in accordance with relevant guidelines and regulations. In particular, written informed consent was obtained from all participants and all HIPAA identifiers were anonymized.

### Equipment and settings

For every patient, one-minute capillary videos were acquired using our optical prototype (Fig. 1). This prototype uses a 5 × Edmund Optics TECHSPEC lens with a Thorlabs DCC3240N camera of 1280 × 1024 pixels, corresponding to a 1360 × 1088 um FOV. Acquisitions were performed at a frame rate 60 frames per second (FPS). The bit depth was 10 bits per pixel in monochrome, the displayed brightness covering the full range of the data. More detailed information on the device components is provided in the Supplementary Methods section “Optical-device components”.

Acquisitions were done at room temperature with optical-coupling oil in the finger well, using the *Labview* software (National Instruments, USA). Raw videos were subsequently *flattened* using the *ImageJ* software (National Institutes of Health, USA), *i*.*e*., the local brightness was normalized through Gaussian filtering to compensate for the effects of non-uniform illumination.

Two units of this prototype were mounted and employed at MGH and HULP, respectively. Following every use on a patient, disinfectant wipes were employed on the device components. The use of this device was approved for clinical research by the Dana-Farber/Harvard Cancer Center protocol #15-070.

### Data collection

For every patient, raw videos were acquired at baseline and during severe neutropenia using our optical prototype. Out of the 26 recruited patients, one dataset was left incomplete because one participant at HULP had a serious adverse event and died of a neutropenia-related sepsis. The complication developed before the second measurement, i.e., before severe neutropenia, and was hence considered unrelated to our study. No other adverse event (serious or non-serious) was reported. For the remaining 25 patients, 6 video datasets were excluded due to insufficient imaging or capillary quality. However, we only analyzed video datasets containing same sets of capillaries at baseline and during severe neutropenia, thus excluding 8 additional patients that contained unpaired capillaries.

Tracking the same capillaries aimed at avoiding bias in the capillary selection, and at minimizing potential confounding factors, *e*.*g*., discrepancies in capillary volume, thus ensuring that changes in event counts best reflect the underlying changes in WBC values between both time points. Note that, while suitable results may be obtained from unpaired capillaries, this remains the topic of further study; conversely, the idea of tracking the same capillaries could be further exploited to devise calibration strategies in the future (see “Discussion”). To follow the same capillaries in a given patient, the optical-prototype user had to locate same capillary areas at both time points. This process was performed manually during live data acquisition using the characteristic capillary distributions and morphologies to identify previously acquired regions. The problem was reduced to a 1D search by choosing areas closely adjacent to the nail boundary.

For each of the 11 compliant datasets, at least one pair of videos—corresponding to acquisitions of one same capillary area in both clinical states—was deemed suitable for further processing. Two distinct capillary regions were used for both Patients 01 and 02. In total, 53 capillaries were selected, followed, and analyzed.

Our study protocol required videos to be acquired within eight hours of a blood test providing reference information. This time window was chosen to maximize the chances that the ground-truth blood-draw measurements would still be valid by the time our data acquisition happened. Accordingly, total-leukocyte, absolute-neutrophil, and lymphocyte concentrations from state-of-the-art blood-cell analytics were obtained for every patient and time point (Table 1). These reference values reveal the sharp decrease in neutrophil concentration between baseline and severe neutropenia. In addition, the reference total-leukocyte values of Table 1 imply that all WBC subtypes are scarce during severe neutropenia, even though the decrease in lymphocyte concentration was less pronounced than for neutrophils.

**Table 1:** Reference values obtained from hospital clinical laboratory.

Patient ID	Leukocytes (cells/µL)		Neutrophils (cells/µL)		Lymphocytes (cells/µL)	
	Baseline	Severe N.	Baseline	Severe N.	Baseline	Severe N.
01	5500	100	4060	10	500	120
02	2000	300	1280	10	420	10
03	5860	210	5660	0	100	110
04	4290	40	2830	20	970	10
05	2640	20	2480	10	130	0
06	2390	100	1840	30	350	0
07	7430	90	7100	0	141	34
08	6370	50	6040	0	150	20
09	3180	40	2770	10	160	20
10	5350	120	3700	0	979	67
11	7760	10	7260	0	357	9

Measurements were carried out using state-of-the-art blood-cell analytics, and the values provided in rounded form. These reference values are also represented graphically in Supplementary Fig. S23. Our study protocol required video data to be acquired within eight hours of the corresponding blood draw.

### Capillary selection

Given the raw videos of the patients, two independent experts selected the capillaries suitable for further analysis based on the following empirical a-priori criteria: (A) Illumination. Capillaries must be visible with sufficient contrast to an observer; (B) Focus. Detailed capillary structures/dynamics must be visible and not blurred out; (C) Flow. Blood flow must exist to allow for potential events to be identified and counted; (D) Stability. Capillaries must fully remain within the video FOV in all frames; (E) Visibility. No object (*e*.*g*., air bubbles) can occlude capillaries. (F) Morphology. Capillaries must exhibit clear arterial and venous limbs; (G) Criteria (A)-(F) must hold in both baseline and severe-neutropenia acquisitions, the goal being to gather paired capillary videos for every patient.

Each expert first evaluated the baseline videos according to Criteria (A)-(F). The sets of candidate capillaries acquired during severe neutropenia were then pre-constrained by the choices already made at baseline. To minimize bias, only those capillaries selected by both raters were included in the study. The resulting capillary-video pairs—at least one per patient—were thus the ones complying with Criteria (A)-(G) according to both experts (Supplementary Figs S1–S13).

### Creation of capillary videos

Based on the raw videos, individual videos were created for all selected capillaries through (a) the definition, on the first video frame, of a rectangular region of interest (ROI) enclosing every selected capillary, using a graphical user interface, and (b) the use of motion compensation (see Supplementary Methods section “Motion compensation of capillary videos”), which locally corrects camera movements and ensures that the position of every capillary remains stable within the corresponding ROI during the whole sequence.

### Event rating

Three independent human raters identified all events in the capillary videos, following specific visual criteria. These criteria stem from existing literature that describes the consequence of the passage of a WBC through a capillary of similar diameter as the creation of a region depleted of RBCs^15,16,33^. Specifically, moving optical-absorption gaps referred to as *events*:

- Are noticeably brighter than the surrounding capillary flow
- Can be identified as clear objects moving along the capillary flow
- Occupy the whole capillary diameter and extend along the flow direction

Raters were blinded with respect to the others and to the blood-analytics, physiological state, patient, and temporal information. This experimental protocol ensured that image analysis was performed without observer bias. Each rater labeled the frames and spatial locations inside the capillary where these events occurred, using a graphical user interface. Following this process, the labeled events could be visualized on the capillary videos or on spatio-temporal (ST) maps (see Supplementary Methods section “Event visualization on videos and ST maps” and Supplementary Fig. S14 for technical details).

The indexing of the videos used by the raters for event identification and counting in every given patient was obtained by randomly shuffling the original-video names, rendering access to the original indexing or (non-)neutropenic state impossible. All capillary videos contained strictly equal amounts of frames. No information in the video content or naming contained patient information.

### Statistical analysis

For event counting, we required the majority of the human raters to agree on an event being observed, based on the labels obtained from all raters. By convention, we assumed that at least *R* raters have jointly marked a given event if the average mark times from at least *R* raters lie within at most ten frames from each other, *i*.*e*., 1/6 second at 60 FPS, which is substantially shorter than average times between consecutive events (Supplementary Fig. S15). Exact spatial overlay of the labels was not required. Counts were obtained accordingly for every capillary and time point, *i*.*e*., summing all majority events from the corresponding videos (Fig. 6).

This approach based on three independent raters mimicked strategies others have used for image-analysis applications^34–38^. For example, a “ground truth” was established for cell-tracking studies by (1) three human experts independently tagging the datasets and (2) using a “majority-vote” mechanism to determine the ground truth for specific cell components being tracked^34–36^. In our case, as in prior literature, this approach using human raters serves as a first step towards developing an algorithmic approach^39^.

To explain further, the rationale for using multiple human raters is that visual evaluation of images or videos is intrinsically subjective and can be particularly sensitive to noise. Differences in the judgment by human raters explain discrepancies between the amounts of events tagged by a majority of raters versus those tagged by an individual rater (see “Results”). Our strategy to reduce noise (*i*.*e*., to reduce the likelihood that a tagged event was just noise) was to adopt the majority-rater approach^34–38^.

At the patient level, majority-event counts were averaged across all available capillaries (Fig. 7). The use of multiple capillaries is beneficial because counting precision in single capillaries (a) is limited by shot noise, which is proportional to the square root of the amount of counts, (b) is limited by potential WBC phenomena not captured by our event-detection criteria and occurring in some capillaries, such as margination^15,33^, (c) depends on the particular capillary morphology and flow rate, and (d) depends on the particular positioning of the capillary in the underlying capillary network^40^. While averaging across capillaries will not fully eliminate the aforementioned sources of variability, it proved sufficient to discriminate between baseline and severe neutropenia (Fig. 7). The beneficial effect of averaging across capillaries was also corroborated by the fact that classification results improved with the amount of capillaries used per patient (Supplementary Fig. S16).

When comparing individual or average capillary-count values between baseline and severe neutropenia, the Wilcoxon two-sided signed-rank test was used on our paired data. The use of this method avoided the statistical assumption that counts are normally distributed while testing the hypothesis *H*_0_ that there is no difference between counts obtained at baseline and during severe neutropenia for the same capillaries. The significance level for this test was set as α = 0.05. Besides this statistical test, receiver-operating-characteristic curves were generated to assess the performance of binary-class classification between baseline and severe neutropenia as a function of a varying threshold count.

### Data availability

The datasets generated during and/or analysed during the current study are available from the corresponding author on reasonable request.

## Results

### Portable optics allows acquiring stable high-speed and high-contrast videos of multiple nailfold capillaries *in-vivo*

We designed a portable and custom optical prototype (Fig. 1) to record high-quality microscopy videos of the microcirculation in human nailfold capillaries, which we employed on the ASCT patients of our study (Fig. 2). A three-dimensional (3D) model of our device is shown in Fig. 1A. The patient’s finger is inserted from the top into the well of a 3D-printed semi-spherical easily sanitized hand rest (Fig. 1B) designed to ergonomically hold the patient’s hand with sufficient stability to record one-minute videos. Capillary videos are acquired from the nailfold region in the patient’s finger (Fig. 1C).

Several features of our prototype were optimized from the perspective of event detection, as opposed to generic off-the-shelf solutions used in prior work such as capillaroscopes. In particular, our device maximizes RBC-to-non-RBC contrast and allows acquiring high-resolution videos at 60 FPS, while providing enhanced stability during acquisition through its hand rest. This allows to track event movement with sufficient temporal resolution, knowing that the range of flow speeds in nailfold capillaries is 100–1,000 µm/s^41,42^. By contrast, the lower frame rate of capillaroscopy videos—typically 15 FPS at high resolution^39^—makes such tracking challenging; events approaching 1,000 µm/s would typically be missed or appear on only one frame.

To minimize the acquisition time per patient, our prototype was also designed to image multiple capillaries within one same FOV (Fig. 3; Supplementary Movie S1). Single acquisitions could contain up to 10 capillaries of suitable quality (Supplementary Figs S1–S13); this could not be achieved when employing off-the-shelf capillaroscopes. Our simple optical approach made the acquisition of multiple capillaries particularly convenient, unlike other techniques proposed for capillary imaging, such as encoded confocal microscopy, which focuses on single capillaries^33^.

The capillaries that we imaged are so narrow, with typical widths of 10–20 microns^27^, that WBCs are forced to squeeze through them one by one. Considering one example capillary (Fig. 4; Supplementary Movie S2), video frames reveal the passage of an event in the microcirculation, which can be perceived as a moving “bright” object of approximately of the same size as the capillary diameter (~15 µm), and whose brightness is substantially higher than the surrounding RBCs.

Event movements could be clearly followed across successive video frames, and, as such, were visually identifiable by a human observer. Accordingly, we instructed three blinded human raters to tag all events in our capillary videos (Fig. 5), following specific visual criteria (see “Methods”). ST maps provide a convenient alternative representation for visualizing all event trajectories with the marks from these raters in the one-minute capillary videos (Supplementary Movie S3). Our empirical observation is that event trajectories visible on the ST map corresponded well with events identified by the raters from the videos (Fig. 5).

### Microcirculation events with specific features constitute usable proxies of WBC

Based on our optical prototype, we recorded and analyzed 22 video datasets from 11 ASCT patients undergoing chemotherapy at two different time points (Fig. 2): pre-chemotherapy baseline (>1,500 neutrophils per µL) and severe neutropenia (<500 neutrophils per µL). The same sets of capillaries were acquired at baseline and during severe neutropenia for every patient (Fig. 3) to ensure the consistency of the results and avoid potential confounding factors (see “Methods”). The choice to acquire one-minute videos was motivated by the need to overcome the shot noise associated with the discrete nature of the events (see Supplementary Methods section “Acquisition time under shot noise”).

In these videos, the consistency with which events were identified by the human raters in the capillary microcirculation depended on whether acquisitions were performed at baseline or during severe neutropenia. In baseline cases, most identified events (62%) were *majority events*, *i*.*e*., events tagged by two or more raters (see “Methods”). In severe-neutropenia cases, only 22% of them were majority events, which was associated with lesser amounts of consistent visual features (Supplementary Fig. S17). This highlights the importance of our majority-rater approach to reduce noise (see “Methods”).

The number of majority events in a capillary acquired during severe neutropenia was consistently lower than in the same capillary acquired during baseline (Fig. 6). Overall, paired capillary counts showed a highly statistically significant difference (Wilcoxon two-sided signed-rank test, *N* = 53, *P* = 5 ⋅ 10^−9^) between baseline and severe neutropenia. However, when considering non-majority events, *i*.*e*., events tagged by single raters, this difference was substantially less significant (Wilcoxon two-sided signed-rank test, *N* = 53, *P* = 0.04) than with majority events (Supplementary Fig. S18).

The above results suggest that events with consistently detectable visual features correlate with the presence of WBC and neutrophils. Majority events were thus treated as proxies to WBCs. Additionally, when diameters were recorded at the capillary segments where majority events were tagged, their distribution fell within the range of WBC diameters, *i*.*e*., 10-20 µm (Supplementary Fig. S19), even though human raters were not instructed to label flow gaps at locations of any specific diameter (see Methods). This provides additional confidence that majority events could be associated with WBCs flowing in single file.

### Counts of microcirculation events allow classifying the baseline state from the severe-neutropenic state non-invasively

Counts from distinct capillaries tended to vary for the same patient, sometimes reaching low values even at baseline. Such variations may be associated with several factors—such as an uneven distribution of WBCs across the capillary network^40^—which motivated the averaging of counts across several capillaries for every patient to increase results robustness (see “Methods”).

When averaging majority-event counts across all capillaries for a given patient and time point, the paired differences in patient counts between the neutropenic and baseline states remained apparent (Fig. 7) and highly statistically significant (Wilcoxon two-sided signed-rank test, *N* = 11, *P* = 0.00097) with a median difference of 30 counts between baseline and severe neutropenia. Counts displayed very little overlap between baseline and severe neutropenia, which allowed for relatively robust classification. Specifically, at a threshold of seven counts, median counts across all capillaries of every given patient could correctly classify all baseline cases and 10 of 11 neutropenic cases. Patient 06 displayed a lower level of discrimination because of three capillaries with low majority-event counts at baseline, particularly in one of them (Supplementary Movie S4; second capillary of Patient 06 from the left in Fig. 6). The misclassification in Patient 10 was associated with a high event count in one capillary at nadir (Fig. 6). This outlier played a critical role because only two capillary pairs were available for this patient.

Majority events yielded substantially better results in patient counts than events marked by single raters (Supplementary Fig. S20), where count differences below statistical significance were obtained between baseline and severe neutropenia (Wilcoxon two-sided signed-rank test, N = 11, *P* = 0.17), with a median difference of only eight counts. The amount of capillaries used per patient positively correlated with the classification performance, with areas under curve of 0.68, 0.84, 0.88, 0.95, and 1.00 obtained for majority-event counts from one, two, three, four and five capillaries—when available—per patient, respectively (Supplementary Fig. S16). The two or three first added capillaries accounted for most gain in classification performance.

These results indicate that, while observations based on single capillaries or single-rater-event counts can yield high rates of erroneous classifications, the averaging of majority-event counts across more than three capillaries per patient allows for a robust discrimination between baseline and severe neutropenia.

## Discussion

The goal of this study was to investigate whether severe neutropenia can be detected non-invasively in humans based on optical imaging. From that perspective, our results demonstrate the relevance of using non-invasive videos of the microcirculation in nailfold capillaries. They also validate our overall classification strategy as a proof of principle towards the long-term objective of non-invasive WBC counting using a portable optical prototype and data analysis.

By design, our clinical study focused on absolute-neutrophil-count levels at baseline (>1,500 neutrophils/µL) and severe neutropenia (<500 neutrophils/µL) in the same patients. While our classification approach was not assessed in the mild (grade II, >500 and <1,500 neutrophils/µL) and moderate (grade III, >500 and <1,000 neutrophils/µL) neutropenia cases, our current observations may be extrapolated to these additional ranges, assuming that event counts vary accordingly. This will have to be confirmed in additional studies with additional data throughout the different grades of neutropenia. Overall, more patients, time points, and ranges will be needed to define the range of detection of differences in cell concentration, before such a device can be deployed. Additional data will also allow to assess performance depending on skin thickness and Fitzpatrick index, and under confounding factors such as clubbing, vasculopathy, thrombophilia, and hemorrhage, to name a few. Potential alterations in capillary morphology after chemotherapy may also constitute a confounding factor. In that regard, prior literature suggests that chemotherapy drugs can cause epithelial damage to vessels in humans^43^ and luminal constrictions in animal models^44^. Within our ability to measure, no statistically significant changes in capillary diameter were observed in our clinical data between before and after chemotherapy (Supplementary Fig. S21). This thus also remains a future topic of investigation.

Our method does not intend to replace the gold standard laboratory tests. It rather serves as a monitoring mechanism to flag high-risk patients, who could be promptly referred to further state-of-the-art blood analysis in case of a positive measurement. This precludes the need for a strict correspondence between the estimates from our method and the golden standard. Meanwhile, as a preliminary observation, the event counts obtained for each patient in our study (Fig. 7) are indeed consistent with the corresponding reference WBC concentrations (Table 1) on average. Indeed, assuming an average blood-flow speed of 800 µm/s and an average capillary diameter of 15 µm^27,41^, the median values of the patient counts for baseline and severe neutropenia (Fig. 7), *i*.*e*., 32.00 and 2.00, yield WBC-concentration estimates of about 3,773 and 236 cells/µL. Both estimates fall within the ranges of the corresponding reference values from the gold-standard laboratory assays (Table 1).

Moving forward, the event rating could be performed automatically on the segmented capillary videos (Supplementary Fig. S14). Such an algorithm could follow approaches proposed to detect objects moving through capillaries^39,45^ or more advanced strategies such as machine learning. Several event features, such as contrast, size, or persistence, could be employed. The accumulation of red cells upstream from flow gaps^15^ could also be leveraged as an additional feature to best distinguish gaps created by the passage of WBCs from mere pockets of plasma. In relation to our study, no direct velocity or flow-rate measurements, *e*.*g*., using a photo-acoustic^46^ technique, were carried out. In further work, flow-rate estimation could be exploited to improve our measurements. In particular, using the same types of prototype and capillary videos as in this study, capillary blood flow could also be estimated algorithmically based on image-processing techniques^47–50^. Flow measurements may then allow discarding capillaries with flow rates that are too low to receive leukocytes^40^ and/or to normalize counts by flow volume. Finally, further refinements on our optical prototype could increase the amounts of capillaries per patient satisfying the quality and consistency criteria required for further analysis (see “Methods”). This will be key for a future translation of this technology into clinical practice, knowing that the current failure rates should be reduced for practical usability. The use of more capillaries per patient is also expected to mitigate the influence of outliers (*e*.*g*., misclassified case in Patient 10) since the amount of analyzed capillaries per patient were shown to positively correlate with the classification performance (Supplementary Fig. S16). Acquiring more videos in one or multiple fingers may also serve this goal in future studies.

The constraint of tracking the same capillaries between consecutive time points for the same patients (see “Methods”) may be relaxed to ease the clinical applicability of the method; this remains a topic for further investigations. Another strategy for future work may be to rather exploit this tracking to calibrate our instrument, based on the count and/or reference blood-draw information obtained at the first time point for every patient, and produce more quantitative WBC assessment. In the context of our study, where the goal was to identify severely neutropenic patients, we observed that eliminating capillaries associated with lower counts at baseline—perhaps due to limited flow or to some inhomogeneous WBC distribution in the capillary network in some cases^40^—further improved the separation and classification between baseline and severe neutropenia (Supplementary Fig. S22). These preliminary results suggest that some capillaries may be more suitable for counting and could be identified based on such strategies. Further studies with more time points and a larger patient sample will be required to determine the validity of these approaches. The current proof-of-concept results obtained in this work do motivate and constitute a basis for further improvements in accuracy and automation, although, as mentioned above, the accuracy of our approach need not match the one of standard blood analyzers, particularly if our approach is used as a companion pre-screening tool.

One extension to this study would be to investigate whether specific WBC ranges can be identified beyond mere screening for severe neutropenia. This could broaden the applicability of our approach not only within the context of chemotherapy, but also to new settings, such as infectious diseases or leukostasis. In addition, non-invasive differential counting may also be achieved. Besides opening up new use cases, differential information could increase accuracy for neutrophil counting, as potential false-positive detections due to other cell subtypes would be avoided. At this point, we can conclude that, despite the potential presence of such false detections, our event-counting procedure yielded satisfactory classification results on our clinical data. In future work, differential counts could be obtained based on techniques and data that are similar to the ones employed in this study because WBC subtypes are known to exhibit distinct optical properties and image features, *e*.*g*., non-granular and granular subtypes correspond to distinct gap lengths and backscattering properties in a capillary^15,33,51^. In that regard, an interesting path for future work is to explore how the length or the frequency of gaps is influenced by the cell size and subtype under consideration.

Overall, this study proved that chemotherapy-induced severe neutropenia can be detected non-invasively through the fingernail with a custom-made portable prototype. It represents the first proof of concept for a technology that could measure an important toxicity of chemotherapy by non-invasive optical means. The automation, replication, and refinement of these results may lead to a new paradigm in the monitoring of cancer patients at risk of severe neutropenia. Furthermore, from a more general standpoint, the proposed imaging technique and conceptual approach could constitute one first step towards non-invasive, *in-vivo* WBC counting.

## Electronic supplementary material

Supplementary Material

Supplementary Movie S1

Supplementary Movie S2

Supplementary Movie S3

Supplementary Movie S4
