# Supplementary material for: Non-invasive detection of severe neutropenia in chemotherapy patients by optical imaging of nailfold microcirculation
Source: Sci Rep. 2018 Mar 28;8:5301. doi: 10.1038/s41598-018-23591-0 (PMC5871877; doi:10.1038/s41598-018-23591-0)
Supplement: Supplementary file 1 — Supplementary Material [file 41598_2018_23591_MOESM1_ESM.pdf]

Non-invasive detection of severe neutropenia in chemotherapy patients by optical imaging of nailfold microcirculation

Aurélien Bourquard[1,2,*], Alberto Pablo-Trinidad[2], Ian Butterworth[1], Álvaro Sánchez-Ferro[1,3], Carolina Cerrato[4], Karem Humala[4], Marta Fabra Urdiola[4], Candice Del Rio[5], Betsy Valles[5], Jason M. Tucker-Schwartz[1], Elizabeth S. Lee[6], Benjamin J. Vakoc[7], Timothy P. Padera[8], María J. Ledesma-Carbayo[2], Yi-Bin Chen[5], Ephraim P. Hochberg[5], Martha L. Gray[1,6], Carlos Castro-González[1,2,*]

[1]Research Laboratory of Electronics, Massachusetts Institute of Technology, Cambridge, MA, USA, [2]Biomedical Image Technologies, Universidad Politécnica de Madrid and CIBER-BBN, Madrid, Spain, [3]Centro Integral en Neurociencias HM CINAC, HM Hospitales, Móstoles, Madrid, Spain, [4]Departamento de Hematología, Hospital Universitario La Paz, Madrid, Spain, [5]Massachusetts General Hospital Cancer Center, Harvard Medical School, Boston, MA, USA, [6]Institute of Medical Engineering and Science, Massachusetts Institute of Technology, Cambridge, MA, USA, [7]Wellman Center for Photomedicine, Harvard Medical School and Massachusetts General Hospital, Boston, MA, USA, [8]Edwin L. Steele Laboratories, Department of Radiation Oncology, Massachusetts General Hospital and Harvard Medical School, Boston, MA, USA.

Supplementary methods

Optical-device components

Our prototype used for video acquisition in ASCT patients consists of the following elements:

1. **Imaging objective**. Edmund Optics TECHSPEC 5X. Its features are a 5X magnification and a maximum numerical aperture of 0.15, reduced through a 3D-printed iris of 2.5 mm diameter to maximize depth of focus, a working distance of 16.2 mm, and a maximum FOV size of 1.8 x 1.32 mm. Its fixed-tube and total lengths are 50 mm and 93.81 mm, respectively. Its height, azimuth, and focal position are manually adjustable.

2. **CMOS camera**. Thorlabs DCC3240N. This camera is mounted to the objective and computer-powered through a USB connection. It comprises a global/rolling shutter. Its FOV is 1280 x 1024 pixels, or 1360 x 1088 um at 5X-magnification, corresponding to a pixel size of 1.0625 x 1.0625 um. Its frame rate is 60 FPS in full frame, which ensures sufficient temporal resolution to detect and track events given the 100-1,000-µm/s range of blood flow speeds in

nailfold capillaries.[41,42] It can reach 229 FPS for FOVs restricted to 320 x 240 pixels. Its bit depth is 10 bits per pixel monochrome.

3. **Illuminators**. Two high-power Luxeon LEDs emit deep-blue light at 420 nm to maximize contrast between RBCs—appearing dark in our videos—and optical-absorption gaps. Each LED emits 161 Lumens at 700 mA using an aspheric collection lens with focal length of 20.1 mm, numerical aperture of 0.6, and an adjustable Thorlabs VA100C collimation slit. Rapid-prototyped LED holders are cage-mounted with heat sinks at ~70-degree inclination from the detection axis.

4. **Power driver**. Drives both LED illuminators continuously at constant DC power level.

5. **Disposable hand rest**. Rigidly mounted 3D-printed platform used to hold the finger in a stable position for at least one-minute imaging. This platform comprises a one-size-fits-all finger well, with sets of additional padding inserts for smaller fingers. Optical-coupling oil (Johnson & Johnson, refractive index of 1.51) remains in the finger well.

6. **Laptop and software**. A laptop connected to the CMOS camera is used for power and video acquisition. Specifically tailored LabView software is used for video acquisition and storage. The output data collected for every patient consists of a set of uncompressed videos with time stamps that provide information on the exact acquisition times associated with each frame.

Motion compensation of capillary videos

Motion compensation was carried out automatically based on a specifically tailored algorithm, both implementations being done in MATLAB. Based on one raw video and one rectangular ROI (see the "Creation of capillary videos" section in the main Methods), we devised an algorithm to produce a motion-corrected capillary video—restricted to the user-specified ROI—using *rigid registration*; more specifically, it aligns all video frames with the first one, assuming that camera movements in the ROI are mere X or Y translations, excluding rotations. While frame movements in the raw video can involve long-range deformations, visual inspection of the registration results proved successful when applied to every capillary ROI separately. As a similarity metric and optimization criterion, our algorithm uses *mutual information*, which is a measure based on information theory that copes with slight contrast changes and guarantees accurate sub-pixel alignment.[R1] Prior to this refined registration process, a coarse frame-pre-alignment step was performed to ensure a suitable initialization, based on cross-correlation analysis of pixel values and spatial gradients.

Event visualization on videos and ST maps

In order to best visualize and highlight the events labeled by the human raters in capillaries, we developed a method to simultaneously visualize the frames of the capillary video of interest and the corresponding ST-intensity profile of the capillary. This method allows events to be visualized both explicitly, *i.e.*, as a moving object through the corresponding video frames, and as a fixed profile in their ST-map representation. In the context of our study, this visualization technique had allowed us to retrospectively analyze the distribution of labeled events in our videos as well as the relevance of majority rater agreement.

The concept of a ST map for capillary-flow visualization was proposed in previous literature.[18,39,R2] It is motivated by the fact that events can be conveniently observed in that representation: events associated with WBCs are expected to appear as thick, high-contrast, sparse, and unidirectional ST trajectories.[R3] These properties also relate to the visual criteria that we defined for our raters, *e.g.*, event brightness. ST maps make our rater-marked events appear as well-defined salient trajectories surrounded by a darker background (Fig. 5).

To create our ST maps, we followed an approach that extracts capillary brightness—as averaged over the cross-section—as a function of time and of the cumulative capillary length, based on segmented capillary boundaries.[39] In order to improve the visualization of event trajectories, we normalized the map values by subtracting their local temporal averages as obtained between 50 frames before and after every time point. We performed the initial capillary-segmentation procedure based on an image extracted from the corresponding registered video. Since the capillary profile may be incomplete in a single video frame due to the presence of absorption gaps in the flow, we chose to extract a temporally integrated image, based on an approach akin to that proposed by Allen *et al.*[R4] for nailfold capillaries, where temporally variable features associated with the capillary flow were also enhanced to maximize contrast between the capillary and its surroundings. This approach relates to the concept of motion-contrast enhancement.[R3] Specifically, the image that we used for segmentation was obtained through the integration of temporal-frequency components whose periods were empirically chosen to lie in the [0.25,1.5]-second interval.

Capillary boundaries were first segmented manually, and then refined with the help of an active-contour technique.[R5] The segmentation of both capillary boundaries was finally automatically resampled so as to include 1,000 points each, such that the center of every point pair at the same index of both boundaries lies on the medial axis of the capillary, where the medial axis is the loci of all circles inscribed in the capillary. Finally, based on this segmentation, separations between the arterial limb, venous limb, and loop of the capillary were defined on a case-to-case basis for visualization (Supplementary Fig. S14).

Determination of the video acquisition time

The video-acquisition time t was set to one minute because it was practically suitable for clinical settings while remaining long enough to allow sampling substantially higher amounts of events

N_e in baseline cases compared to severe-neutropenia cases. Even under the shot noise that originates from the quantized nature of events, the count distributions associated with both cases are expected to be disjoint within at least one standard deviation of their means N_b and N_n, respectively, as expected from the calculations detailed below.

To validate this choice, we assumed a worst-case scenario, using a lower-limit case for baseline (C_b = 1,500 neutrophils/µL) and a higher-limit case for severe neutropenia (C_n = 500 neutrophils/µL), which is most difficult to discriminate since the difference between cell concentrations C from both categories is minimized. Typical values from the literature[27,41] were then assumed for capillary diameter (D = 15 µm) and flow speed (v = 800 µm/s), which allowed estimating expected amounts of events from concentrations.[33,39] Specifically, $N_e = \pi \cdot (D/2)^2 \cdot C \cdot v \cdot t$, which yields N_b = 4.24 and N_n = 12.72. Finally, the shot-noise statistics imply that counts vary around these means with standard deviations $N_b^{1/2}$ = 2.06 and $N_n^{1/2}$ = 3.57, respectively. This resulted in count ranges allowing for clear discrimination (Supplementary Fig. S15).

Supplementary figures

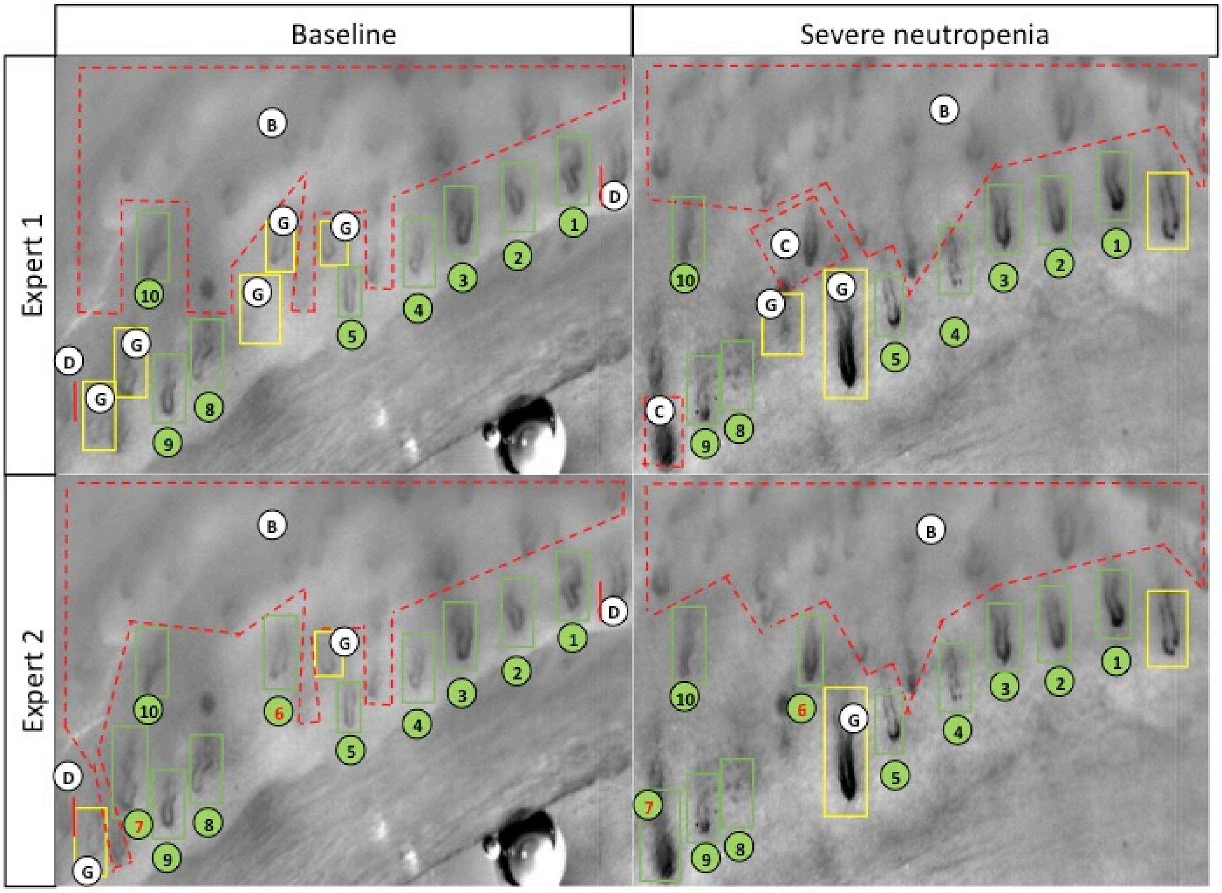

Fig. S1. Capillary selection from both experts in raw-video pair from Patient 01, region 1. The videos acquired in baseline and severe-neutropenic states are displayed. The green boxes outline the selected capillary pairs which, according to each of the two experts complied with the quality criteria for both baseline and severe-neutropenia acquisitions. The red regions/capillaries are discarded due to non-compliance with the quality criteria, *i.e.*, due in this instance to (B) lack of focus, (C) lack of blood flow, and (D) out-of-FOV movements. The red lines/corners in the baseline videos outline the effective FOV outside which capillaries must be discarded as they disappear during several frames due to camera movements during acquisition. The yellow boxes outline capillaries that were initially selected in baseline but were discarded later due to non-compliance in the severe-neutropenia acquisition (G). Both experts made the selection process independently and, after that, only capillaries where both experts agreed were kept (black numbers), discarding the rest of them (red numbers).

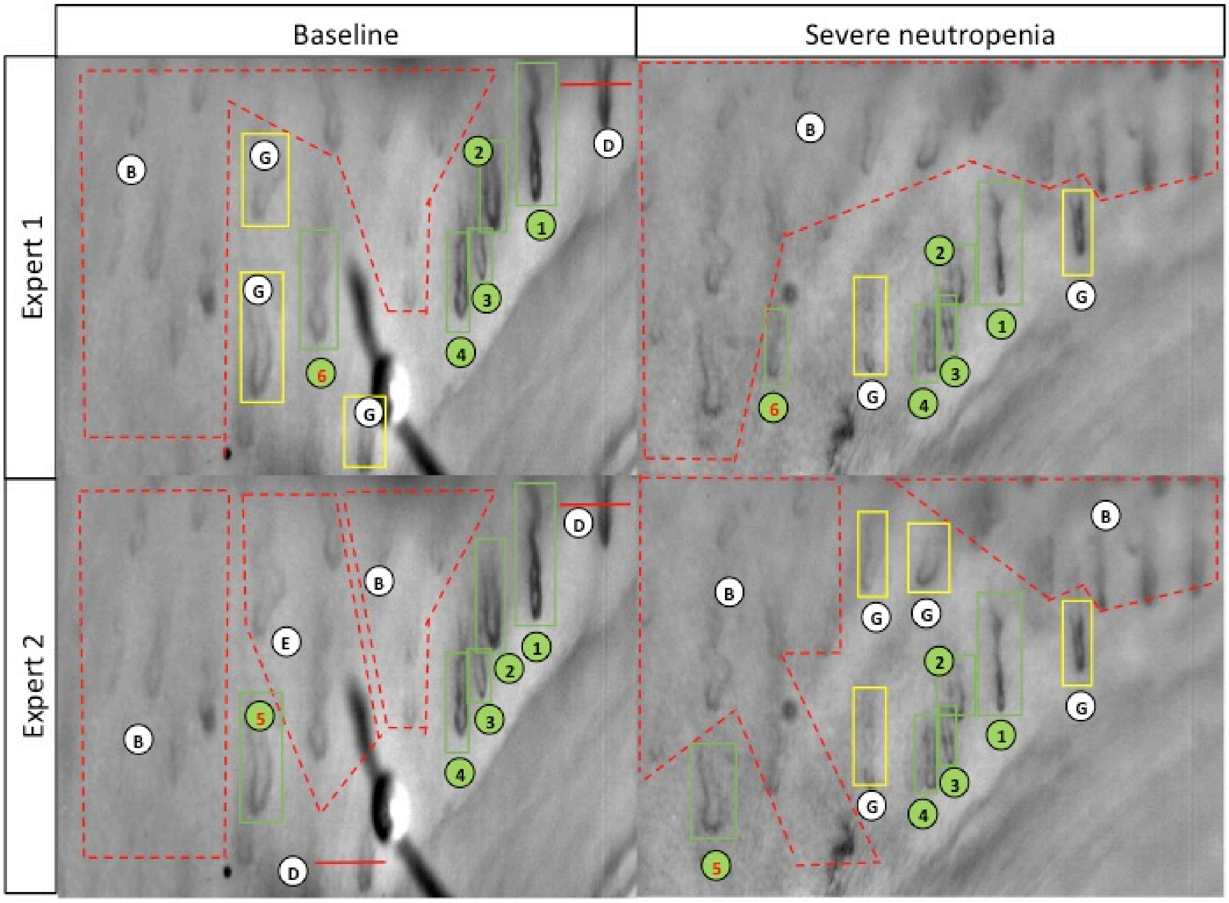

Fig. S2. Capillary selection from both experts in raw-video pair from Patient 01, region 2. The videos acquired in baseline and severe-neutropenic states are displayed. The green boxes outline the selected capillary pairs which, according to each of the two experts complied with the quality criteria for both baseline and severe-neutropenia acquisitions. The red regions/capillaries are discarded due to non-compliance with the quality criteria, *i.e.*, due in this instance to (B) lack of focus, (D) out-of-FOV movements, and (E) occlusions. The red lines/corners in the baseline videos outline the effective FOV outside which capillaries must be discarded as they disappear during several frames due to camera movements during acquisition. The yellow boxes outline capillaries that were initially selected in baseline but were discarded later due to non-compliance in the severe-neutropenia acquisition (G). Both experts made the selection process independently and, after that, only capillaries where both experts agreed were kept (black numbers), discarding the rest of them (red numbers).

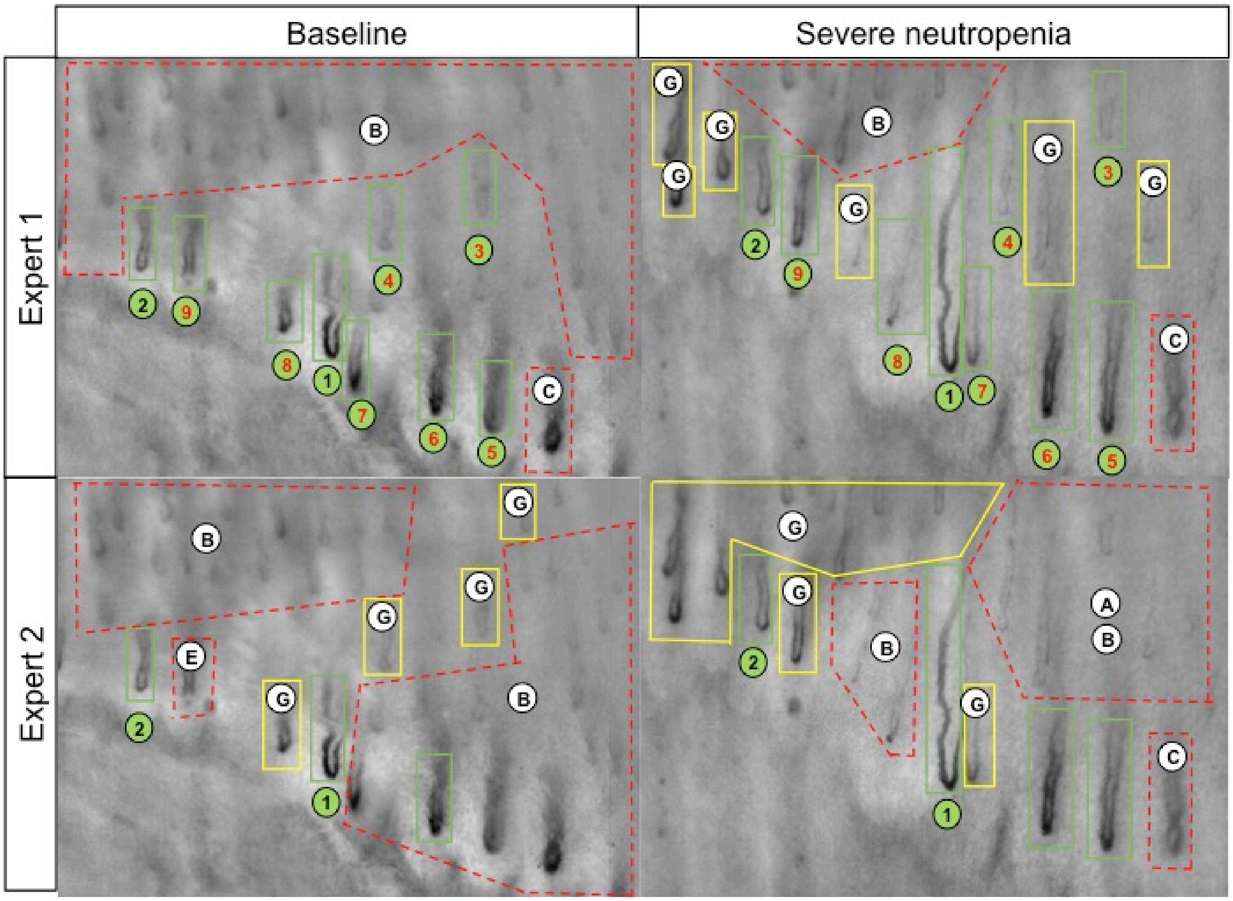

Fig. S3. Capillary selection from both experts in raw-video pair from Patient 02, region 1. The videos acquired in baseline and severe-neutropenic states are displayed. The green boxes outline the selected capillary pairs which, according to each of the two experts complied with the quality criteria for both baseline and severe-neutropenia acquisitions. The red regions/capillaries are discarded due to non-compliance with the quality criteria, *i.e.*, due in this instance to (A) poor illumination, (B) lack of focus, (C) lack of blood flow, and (E) occlusions. The red lines/corners in the baseline videos outline the effective FOV outside which capillaries must be discarded as they disappear during several frames due to camera movements during acquisition. The yellow boxes outline capillaries that were initially selected in baseline but were discarded later due to non-compliance in the severe-neutropenia acquisition (G). Both experts made the selection process independently and, after that, only capillaries where both experts agreed were kept (black numbers), discarding the rest of them (red numbers).

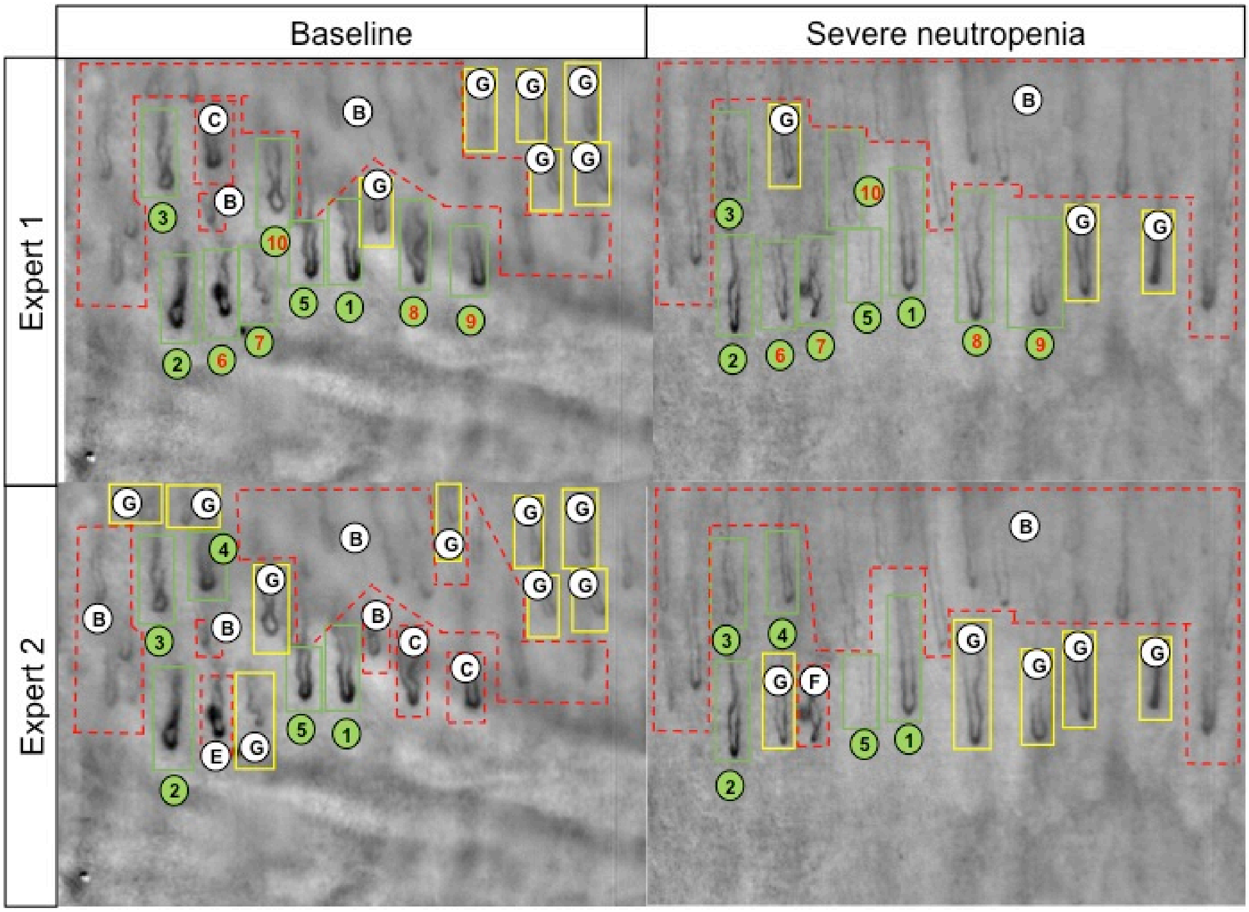

Fig. S4. Capillary selection from both experts in raw-video pair from Patient 02, region 2. The videos acquired in baseline and severe-neutropenic states are displayed. The green boxes outline the selected capillary pairs which, according to each of the two experts complied with the quality criteria for both baseline and severe-neutropenia acquisitions. The red regions/capillaries are discarded due to non-compliance with the quality criteria, *i.e.*, due in this instance to (B) lack of focus, (C) lack of blood flow, (E) occlusions, and (F) lack of clear morphology. The red lines/corners in the baseline videos outline the effective FOV outside which capillaries must be discarded as they disappear during several frames due to camera movements during acquisition. The yellow boxes outline capillaries that were initially selected in baseline but were discarded later due to non-compliance in the severe-neutropenia acquisition (G). Both experts made the selection process independently and, after that, only capillaries where both experts agreed were kept (black numbers), discarding the rest of them (red numbers).

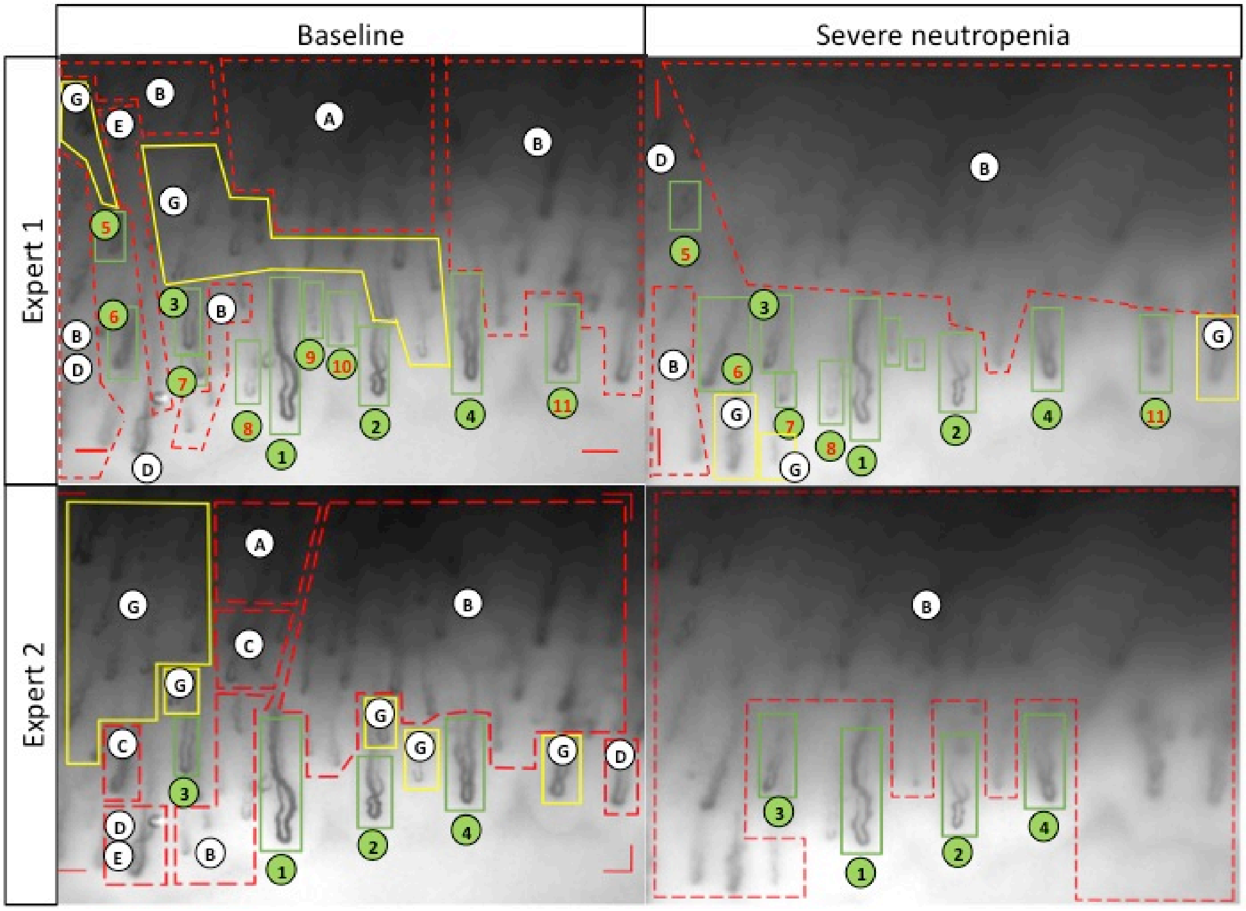

Fig. S5. Capillary selection from both experts in raw-video pair from Patient 03. The videos acquired in baseline and severe-neutropenic states are displayed. The green boxes outline the selected capillary pairs which, according to each of the two experts complied with the quality criteria for both baseline and severe-neutropenia acquisitions. The red regions/capillaries are discarded due to non-compliance with the quality criteria, *i.e.*, due in this instance to (A) poor illumination, (B) lack of focus, (C) lack of blood flow, (D) out-of-FOV movements, and (E) occlusions. The red lines/corners in the baseline videos outline the effective FOV outside which capillaries must be discarded as they disappear during several frames due to camera movements during acquisition. The yellow boxes outline capillaries that were initially selected in baseline but were discarded later due to non-compliance in the severe-neutropenia acquisition (G). Both experts made the selection process independently and, after that, only capillaries where both experts agreed were kept (black numbers), discarding the rest of them (red numbers).

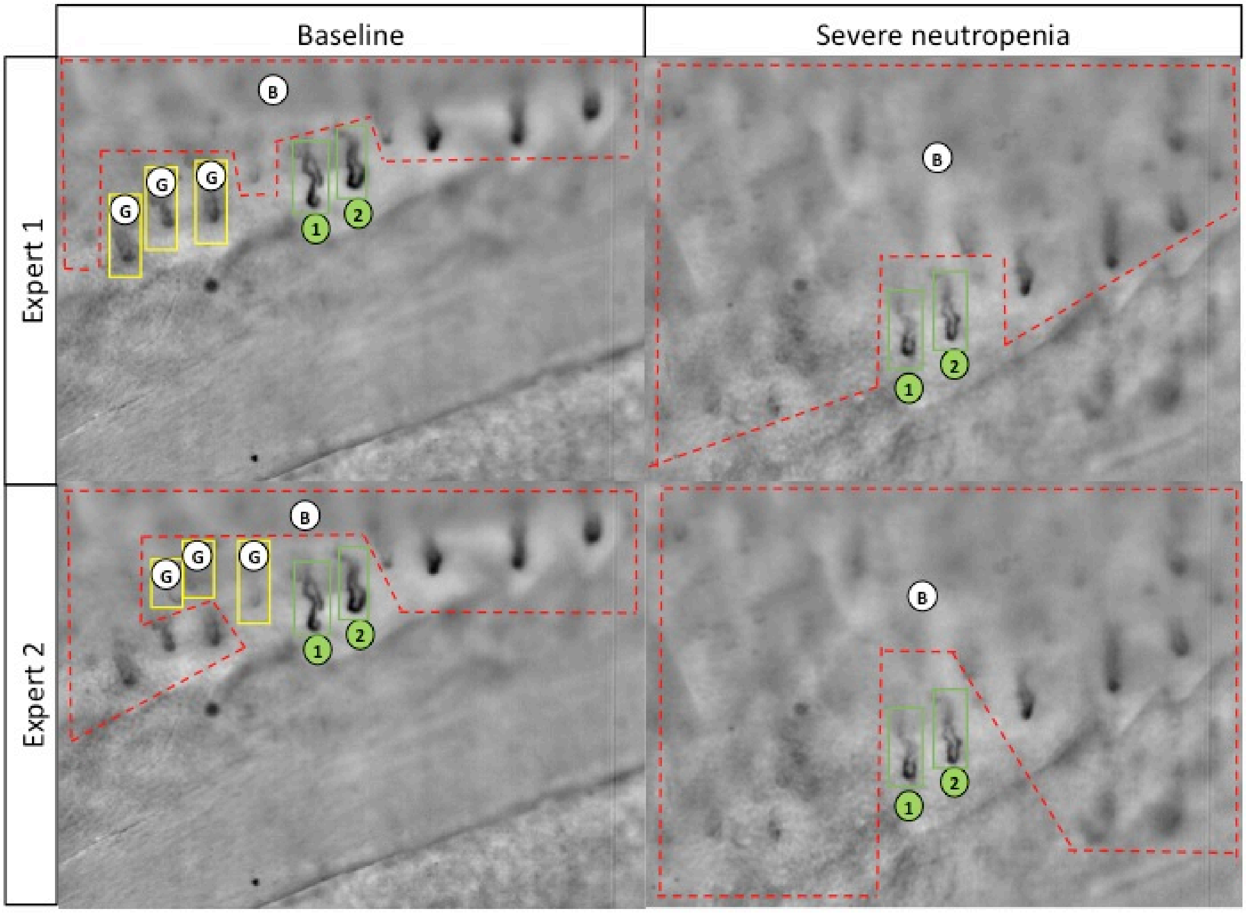

Fig. S6. Capillary selection from both experts in raw-video pair from Patient 04. The videos acquired in baseline and severe-neutropenic states are displayed. The green boxes outline the selected capillary pairs which, according to each of the two experts complied with the quality criteria for both baseline and severe-neutropenia acquisitions. The red regions/capillaries are discarded due to non-compliance with the quality criteria, *i.e.*, due in this instance to (B) lack of focus. The red lines/corners in the baseline videos outline the effective FOV outside which capillaries must be discarded as they disappear during several frames due to camera movements during acquisition. The yellow boxes outline capillaries that were initially selected in baseline but were discarded later due to non-compliance in the severe-neutropenia acquisition (G). Both experts made the selection process independently and, after that, only capillaries where both experts agreed were kept (black numbers), discarding the rest of them (red numbers).

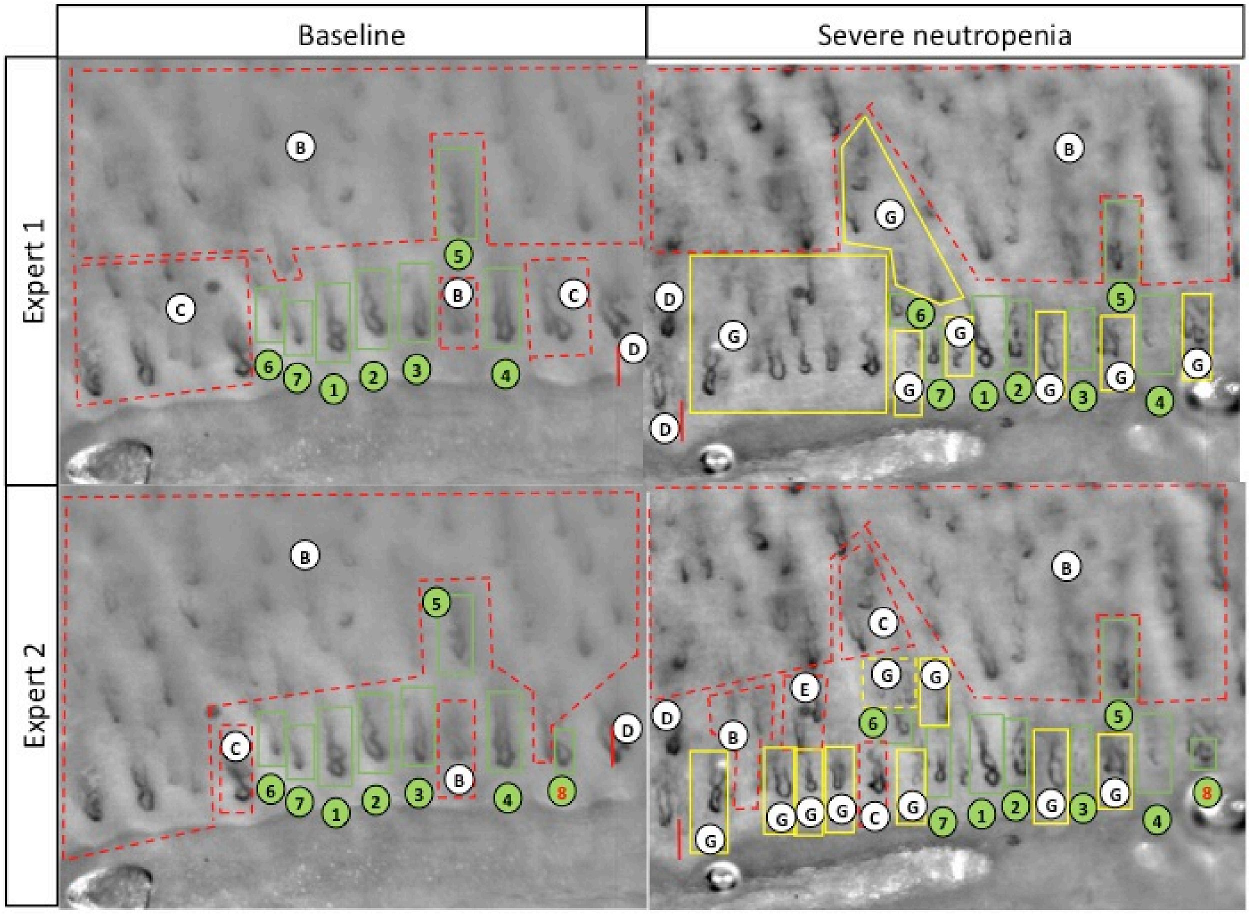

Fig. S7. Capillary selection from both experts in raw-video pair from Patient 05. The videos acquired in baseline and severe-neutropenic states are displayed. The green boxes outline the selected capillary pairs which, according to each of the two experts complied with the quality criteria for both baseline and severe-neutropenia acquisitions. The red regions/capillaries are discarded due to non-compliance with the quality criteria, *i.e.*, due in this instance to (B) lack of focus, (C) lack of blood flow, (D) out-of-FOV movements, and (E) occlusions. The red lines/corners in the baseline videos outline the effective FOV outside which capillaries must be discarded as they disappear during several frames due to camera movements during acquisition. The yellow boxes outline capillaries that were initially selected in baseline but were discarded later due to non-compliance in the severe-neutropenia acquisition (G). Both experts made the selection process independently and, after that, only capillaries where both experts agreed were kept (black numbers), discarding the rest of them (red numbers).

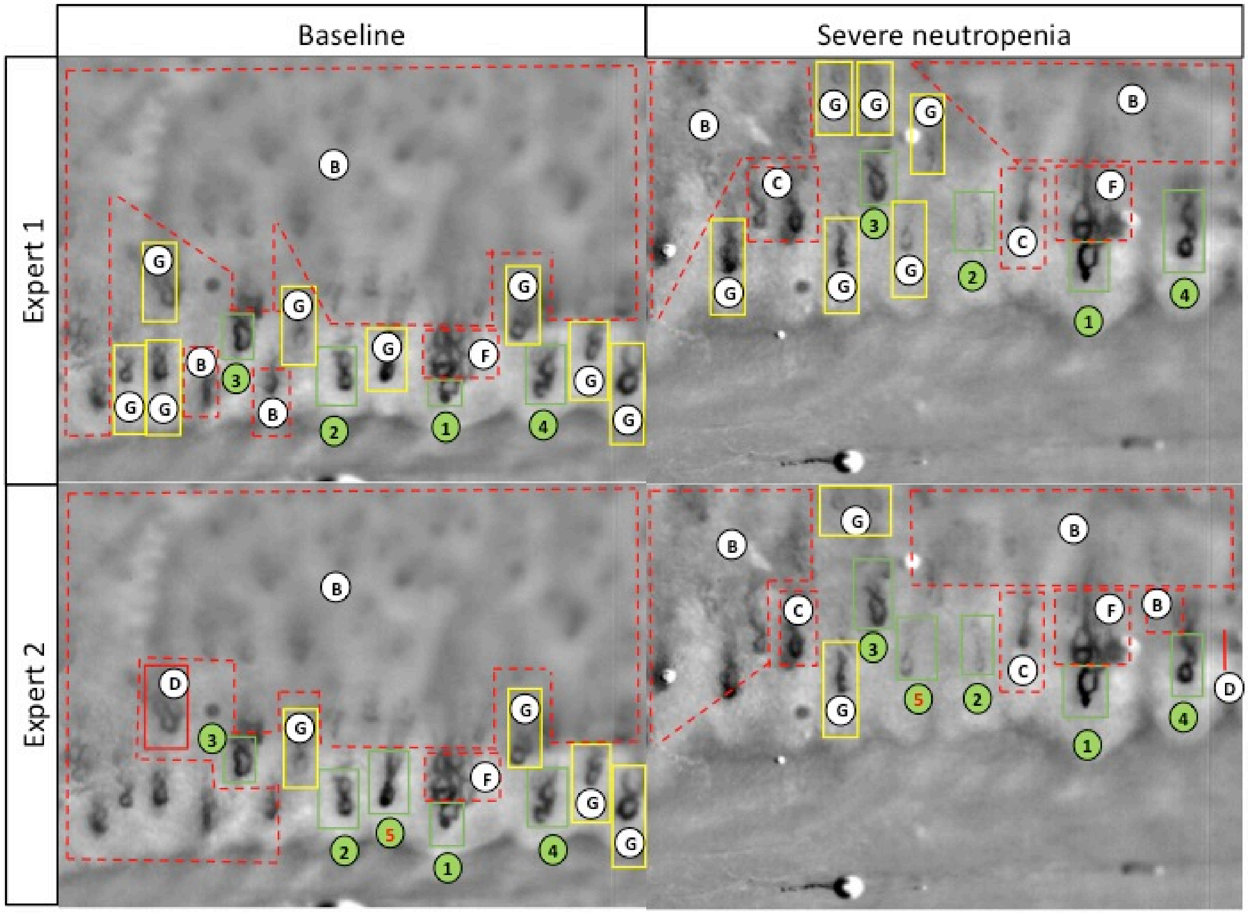

Fig. S8. Capillary selection from both experts in raw-video pair from Patient 06. The videos acquired in baseline and severe-neutropenic states are displayed. The green boxes outline the selected capillary pairs which, according to each of the two experts complied with the quality criteria for both baseline and severe-neutropenia acquisitions. The red regions/capillaries are discarded due to non-compliance with the quality criteria, *i.e.*, due in this instance to (B) lack of focus, (C) lack of blood flow, (D) out-of-FOV movements, and (F) lack of clear morphology. The red lines/corners in the baseline videos outline the effective FOV outside which capillaries must be discarded as they disappear during several frames due to camera movements during acquisition. The yellow boxes outline capillaries that were initially selected in baseline but were discarded later due to non-compliance in the severe-neutropenia acquisition (G). Both experts made the selection process independently and, after that, only capillaries where both experts agreed were kept (black numbers), discarding the rest of them (red numbers).

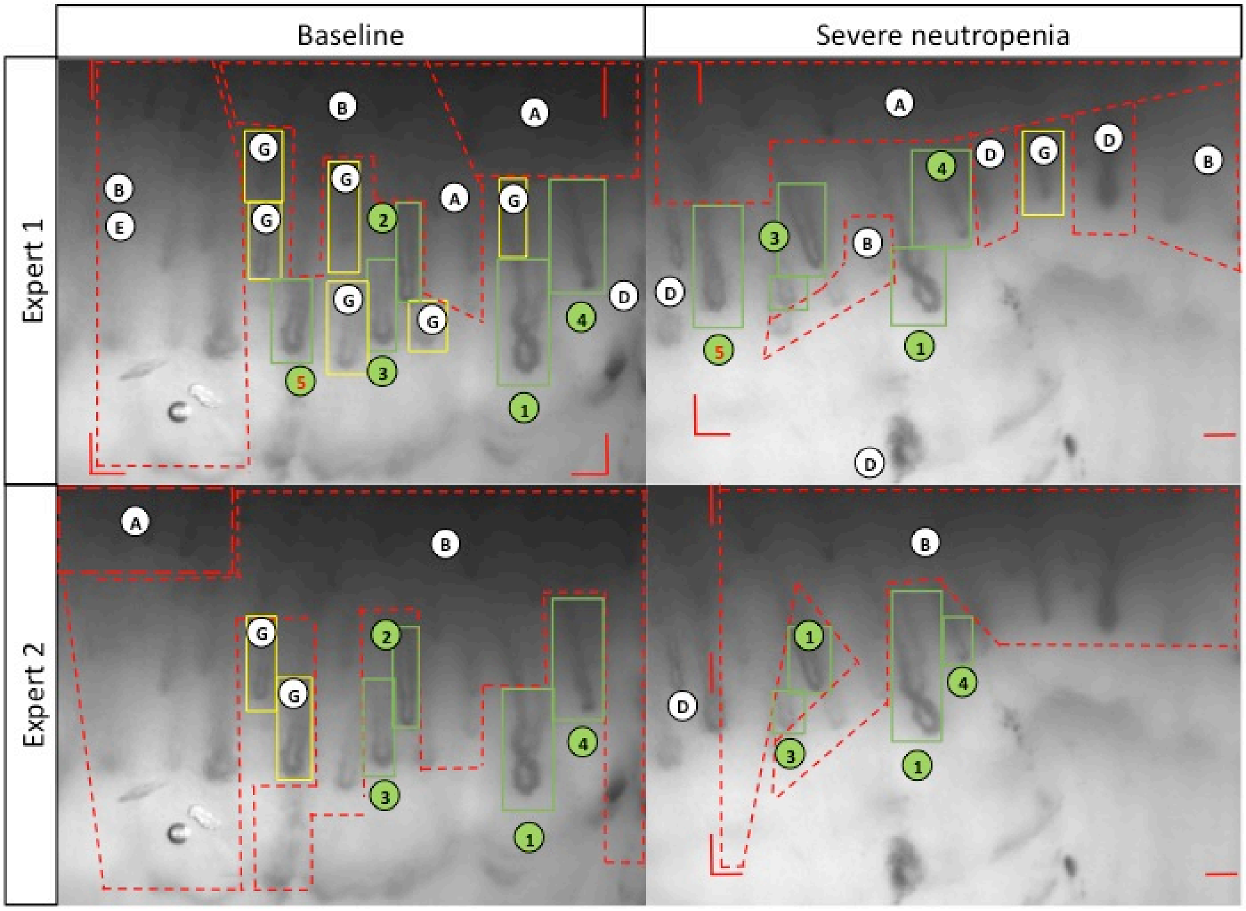

Fig. S9. Capillary selection from both experts in raw-video pair from Patient 07. The videos acquired in baseline and severe-neutropenic states are displayed. The green boxes outline the selected capillary pairs which, according to each of the two experts complied with the quality criteria for both baseline and severe-neutropenia acquisitions. The red regions/capillaries are discarded due to non-compliance with the quality criteria, *i.e.*, due in this instance to (A) poor illumination, (B) lack of focus, (D) out-of-FOV movements, and (E) occlusions. The red lines/corners in the baseline videos outline the effective FOV outside which capillaries must be discarded as they disappear during several frames due to camera movements during acquisition. The yellow boxes outline capillaries that were initially selected in baseline but were discarded later due to non-compliance in the severe-neutropenia acquisition (G). Both experts made the selection process independently and, after that, only capillaries where both experts agreed were kept (black numbers), discarding the rest of them (red numbers).

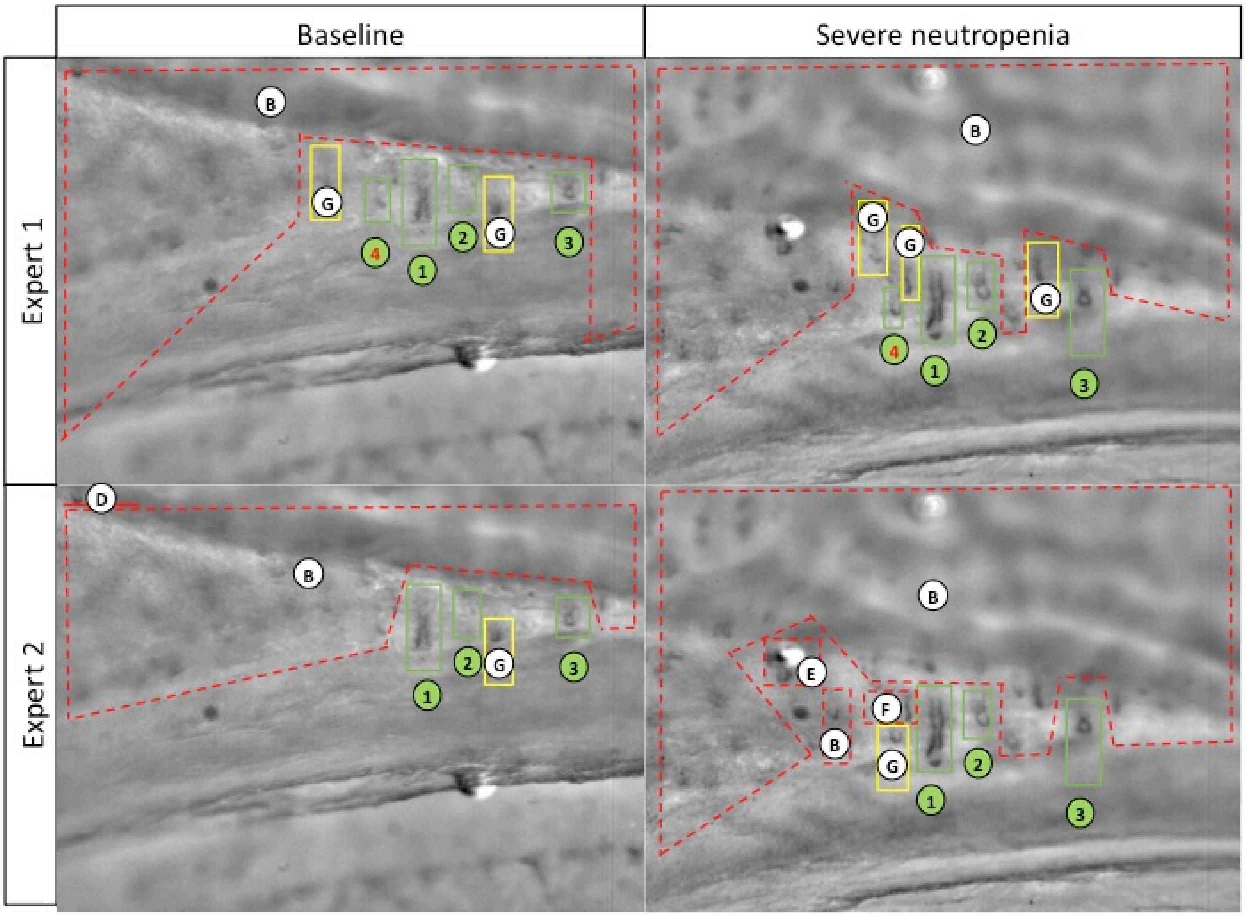

Fig. S10. Capillary selection from both experts in raw-video pair from Patient 08. The videos acquired in baseline and severe-neutropenic states are displayed. The green boxes outline the selected capillary pairs which, according to each of the two experts complied with the quality criteria for both baseline and severe-neutropenia acquisitions. The red regions/capillaries are discarded due to non-compliance with the quality criteria, *i.e.*, due in this instance to (B) lack of focus, (D) out-of-FOV movements, (E) occlusions, and (F) lack of clear morphology. The red lines/corners in the baseline videos outline the effective FOV outside which capillaries must be discarded as they disappear during several frames due to camera movements during acquisition. The yellow boxes outline capillaries that were initially selected in baseline but were discarded later due to non-compliance in the severe-neutropenia acquisition (G). Both experts made the selection process independently and, after that, only capillaries where both experts agreed were kept (black numbers), discarding the rest of them (red numbers).

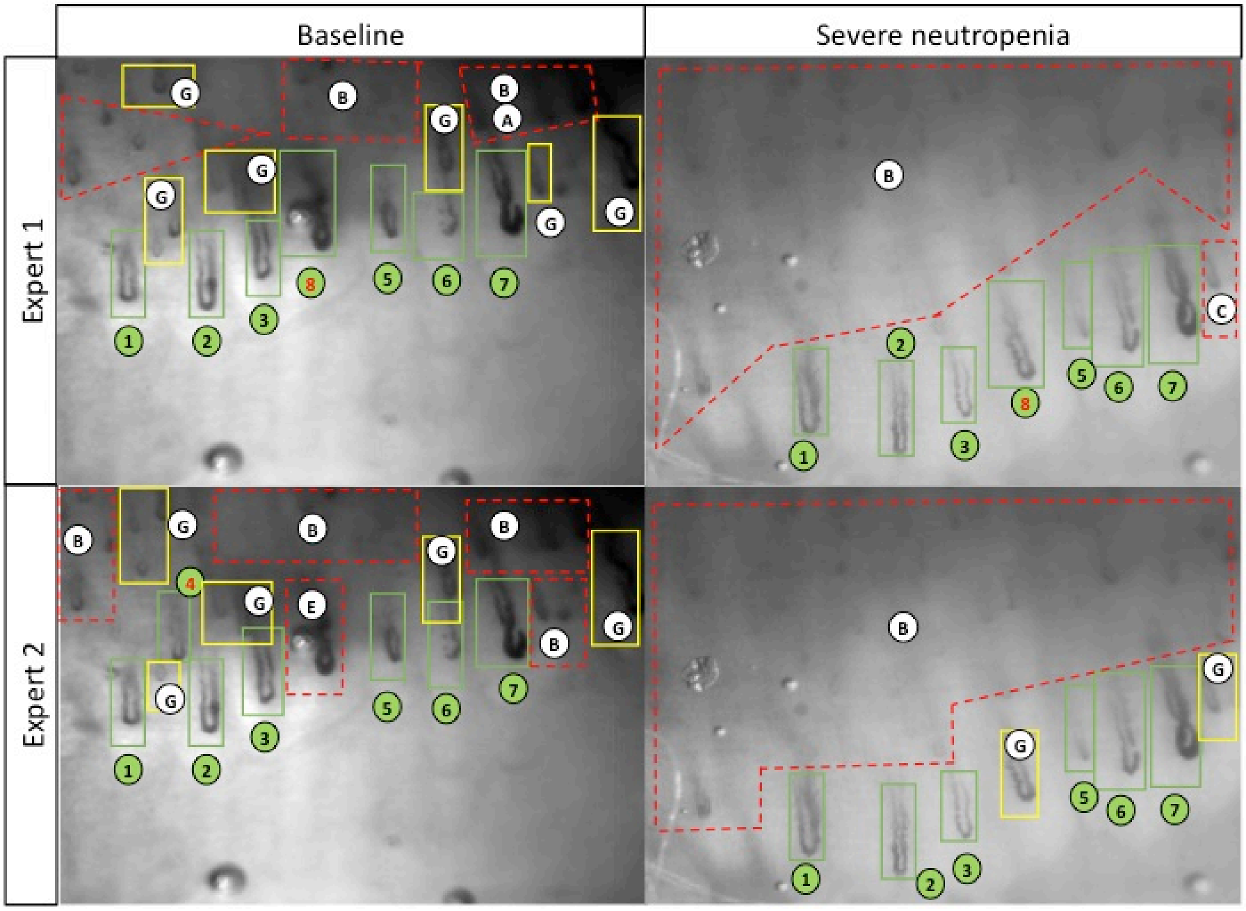

Fig. S11. Capillary selection from both experts in raw-video pair from Patient 09. The videos acquired in baseline and severe-neutropenic states are displayed. The green boxes outline the selected capillary pairs which, according to each of the two experts complied with the quality criteria for both baseline and severe-neutropenia acquisitions. The red regions/capillaries are discarded due to non-compliance with the quality criteria, *i.e.*, due in this instance to (A) poor illumination, (B) lack of focus, (C) lack of blood flow, and (E) occlusions. The red lines/corners in the baseline videos outline the effective FOV outside which capillaries must be discarded as they disappear during several frames due to camera movements during acquisition. The yellow boxes outline capillaries that were initially selected in baseline but were discarded later due to non-compliance in the severe-neutropenia acquisition (G). Both experts made the selection process independently and, after that, only capillaries where both experts agreed were kept (black numbers), discarding the rest of them (red numbers).

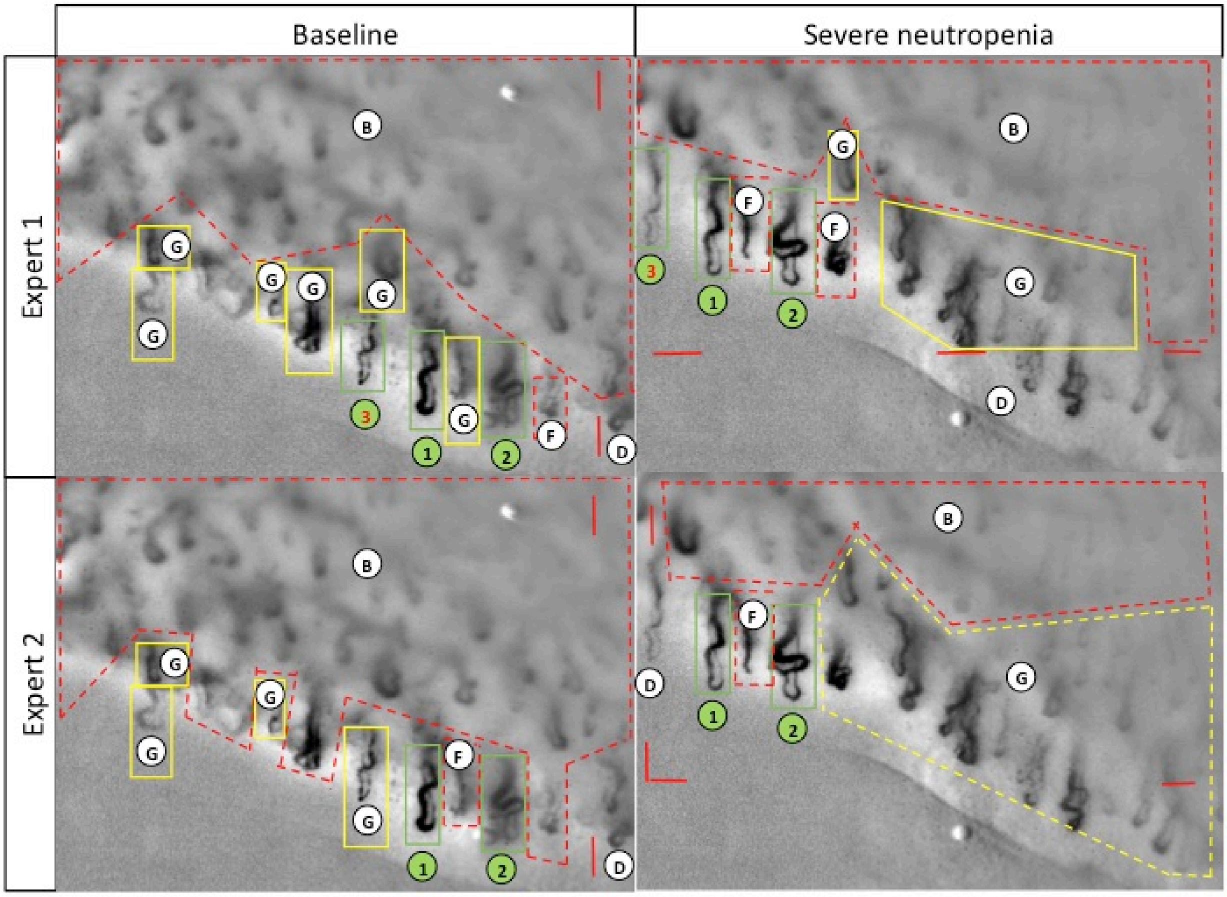

Fig. S12. Capillary selection from both experts in raw-video pair from Patient 10. The videos acquired in baseline and severe-neutropenic states are displayed. The green boxes outline the selected capillary pairs which, according to each of the two experts complied with the quality criteria for both baseline and severe-neutropenia acquisitions. The red regions/capillaries are discarded due to non-compliance with the quality criteria, *i.e.*, due in this instance to (B) lack of focus, (D) out-of-FOV movements, and (F) lack of clear morphology. The red lines/corners in the baseline videos outline the effective FOV outside which capillaries must be discarded as they disappear during several frames due to camera movements during acquisition. The yellow boxes outline capillaries that were initially selected in baseline but were discarded later due to non-compliance in the severe-neutropenia acquisition (G). Both experts made the selection process independently and, after that, only capillaries where both experts agreed were kept (black numbers), discarding the rest of them (red numbers).

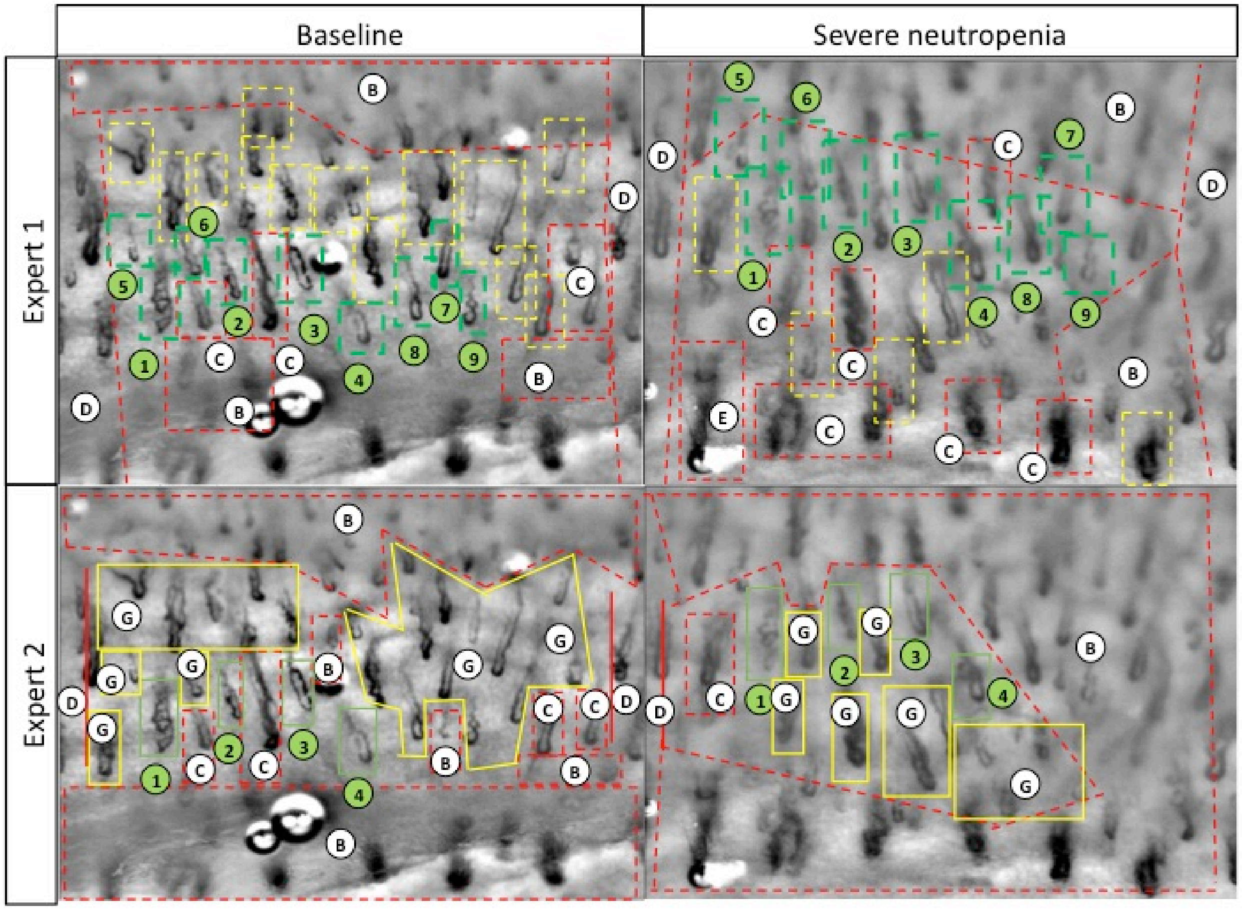

Fig. S13. Capillary selection from both experts in raw-video pair from Patient 11. The videos acquired in baseline and severe-neutropenic states are displayed. The green boxes outline the selected capillary pairs which, according to each of the two experts complied with the quality criteria for both baseline and severe-neutropenia acquisitions. The red regions/capillaries are discarded due to non-compliance with the quality criteria, *i.e.*, due in this instance to (B) lack of focus, (C) lack of blood flow, (D) out-of-FOV movements, and (E) occlusions. The red lines/corners in the baseline videos outline the effective FOV outside which capillaries must be discarded as they disappear during several frames due to camera movements during acquisition. The yellow boxes outline capillaries that were initially selected in baseline but were discarded later due to non-compliance in the severe-neutropenia acquisition (G). Both experts made the selection process independently and, after that, only capillaries where both experts agreed were kept (black numbers).

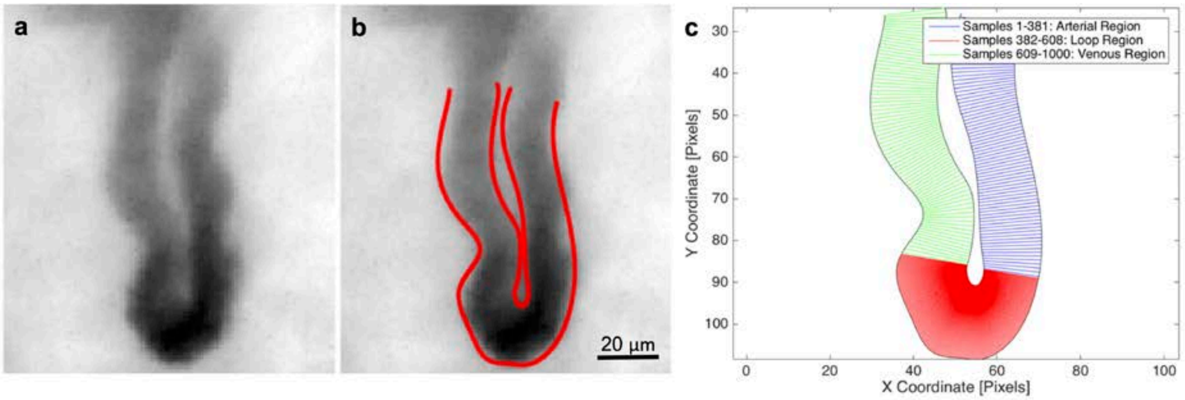

Fig. S14. Example of a capillary segmentation. (a) Capillary from patient 02. **(b)** Same capillary with supervised segmentation (red). **(c)** Separations between arterial limb (green), venous limb (blue), and loop (red) of the capillary can be defined on a case-to-case basis for visualization.

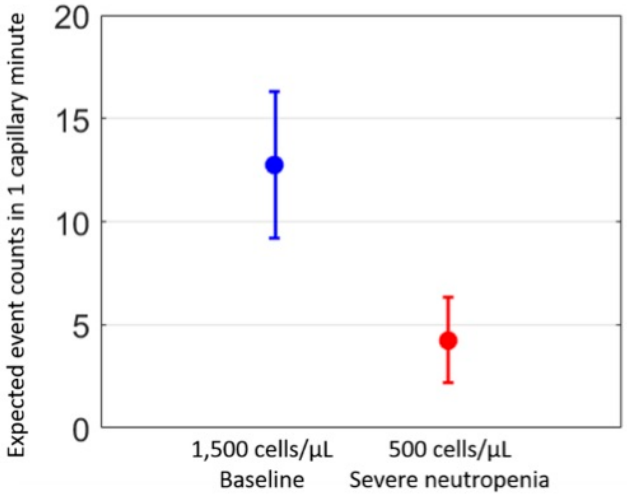

Fig. S15. Expected amounts of events per capillary minute under shot noise. Assuming typical capillary-diameter and speed values from the literature, amounts of expected events at baseline (blue; 1,500 neutrophils/µL) clearly exceed the corresponding amounts during severe neutropenia (red; 500 neutrophils/µL) for a single minute of acquisition, even under shot noise. Shown are expected count averages (central dots) along with expected variations originating from shot noise up to one standard deviation (bars).

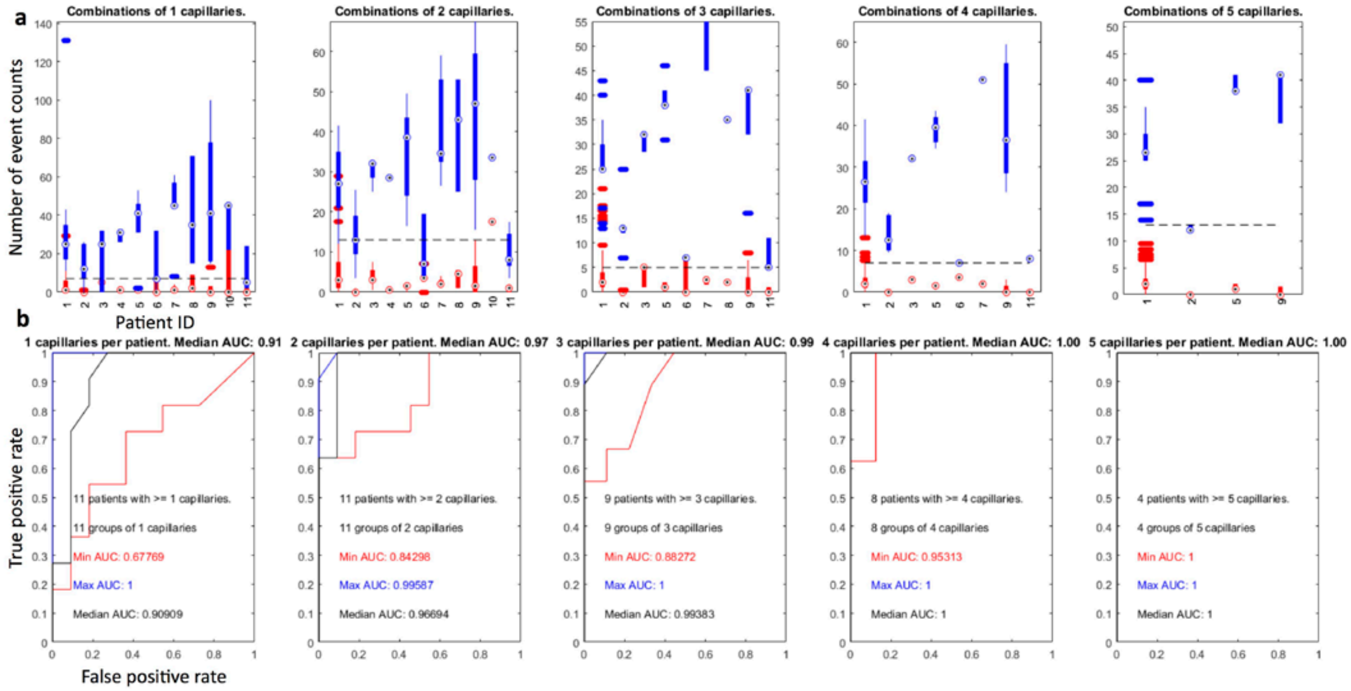

Fig. S16. Discrimination between baseline and severe neutropenia using distinct amounts of capillaries per patient. (a) Number of event counts shown for baseline (blue dots) and severe neutropenia (red dots) resulting from averaging across $N = 1, 2, 3, 4, 5$ capillaries per patient, when available. The corresponding cross-capillary variability is also shown for each patient (blue and red bars with notch extremes determined as $q_2 \pm 1.57(q_3 - q_1) / N^{1/2}$, where the q_i are the respective quartiles, and where N is the amount of available paired data points). For every fixed amount N of capillaries per patient to be assessed, results were averaged over 10,000 trials with randomly picked sets of capillaries, so as to handle amounts of cross-patient capillary combinations that are otherwise intractable. **(b)** Receiver-operating-characteristic curves for classification of baseline vs. severe neutropenia based on integrating $N = 1, 2, 3, 4, 5$ capillaries per patient. The patient-level distributions of the resulting count values show that their discriminatory power increases with the amount of combined capillaries per patient. Specifically, the minimum area under curve consistently increases with the amount of combined capillaries from 0.68 to 0.84, 0.88, 0.95 and 1.00 when including 1, 2, 3, 4 and 5 capillaries, respectively.

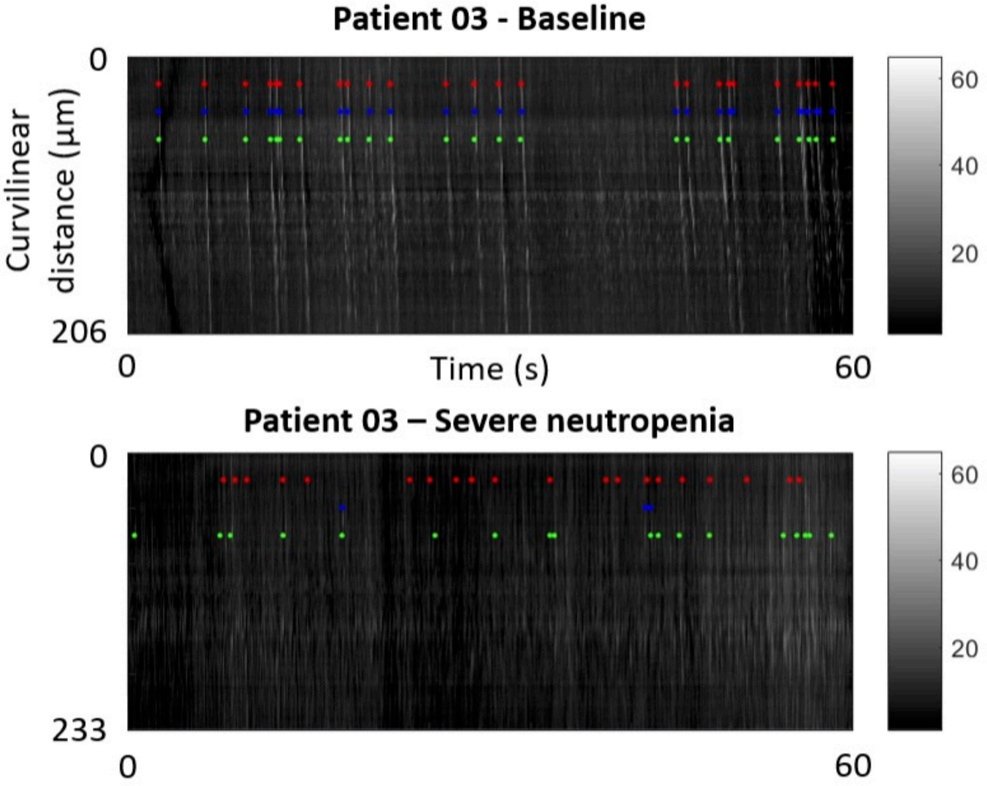

Fig. S17. ST maps of capillaries with high versus low ratio ratios of majority events. For each event, the first click from each of the three human raters is displayed with a red, blue, and green dot, respectively. Inter-rater agreement was higher in baseline capillaries (top map) compared to the case of severe neutropenia (bottom map), indicating that events in baseline correspond to more consistently observable physical phenomena.

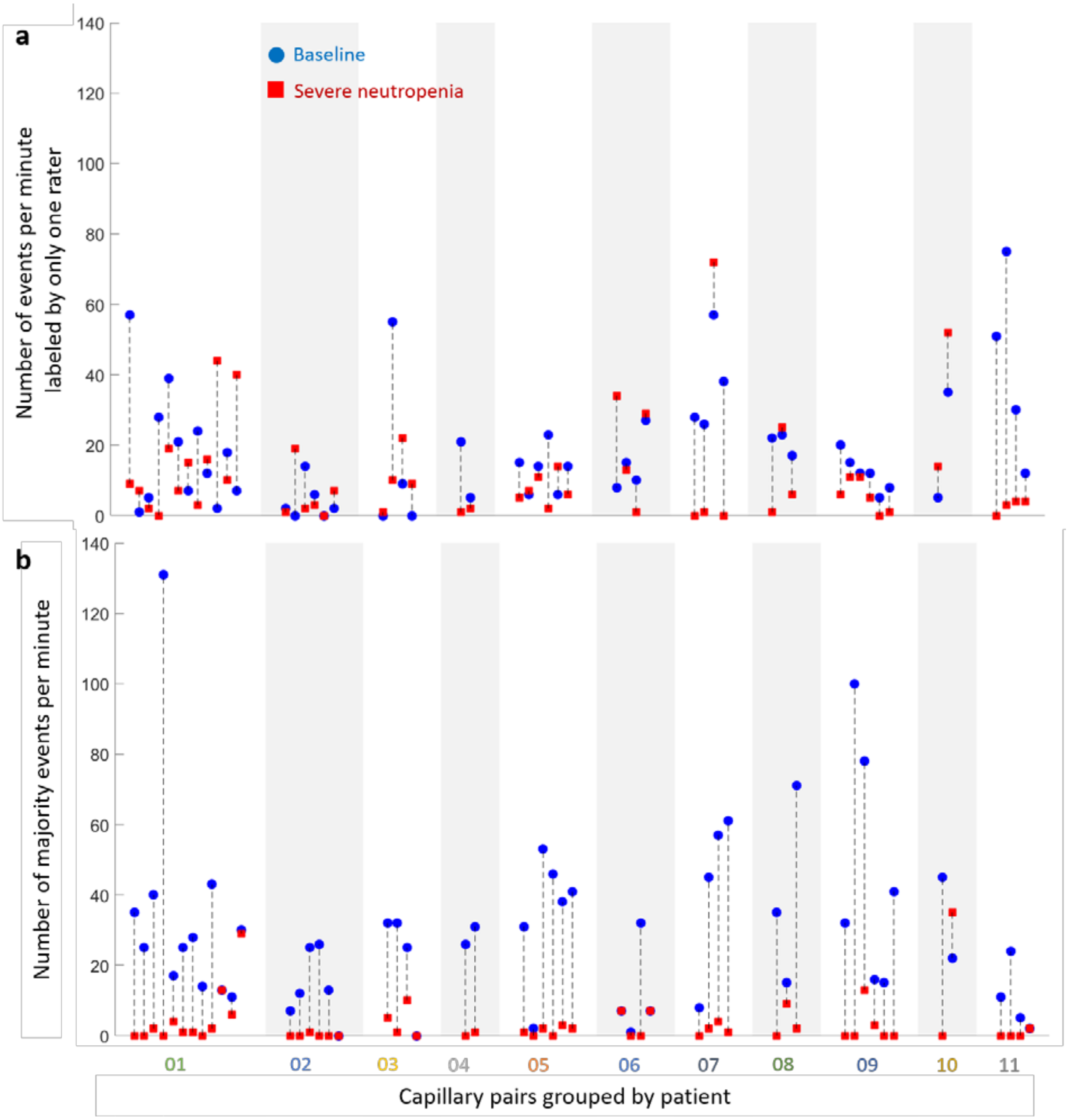

Fig. S18. Numbers of single-rater and majority events per minute in all studied capillary pairs. (a) Counts of single-rater events. **(b)** Counts of majority-rater events. Differences in capillary counts between baseline (blue dots) and severe neutropenia (red squares) displayed a substantially lower statistical significance in the single-rater case (Wilcoxon signed-rank test, N=53, P=0.04) than when based on majority events ($P=5 \cdot 10^{-9}$). This indicates that events labeled by single raters are more prone to noise and contain less information than those where multiple raters agreed. Capillary pairs are grouped by patient ID.

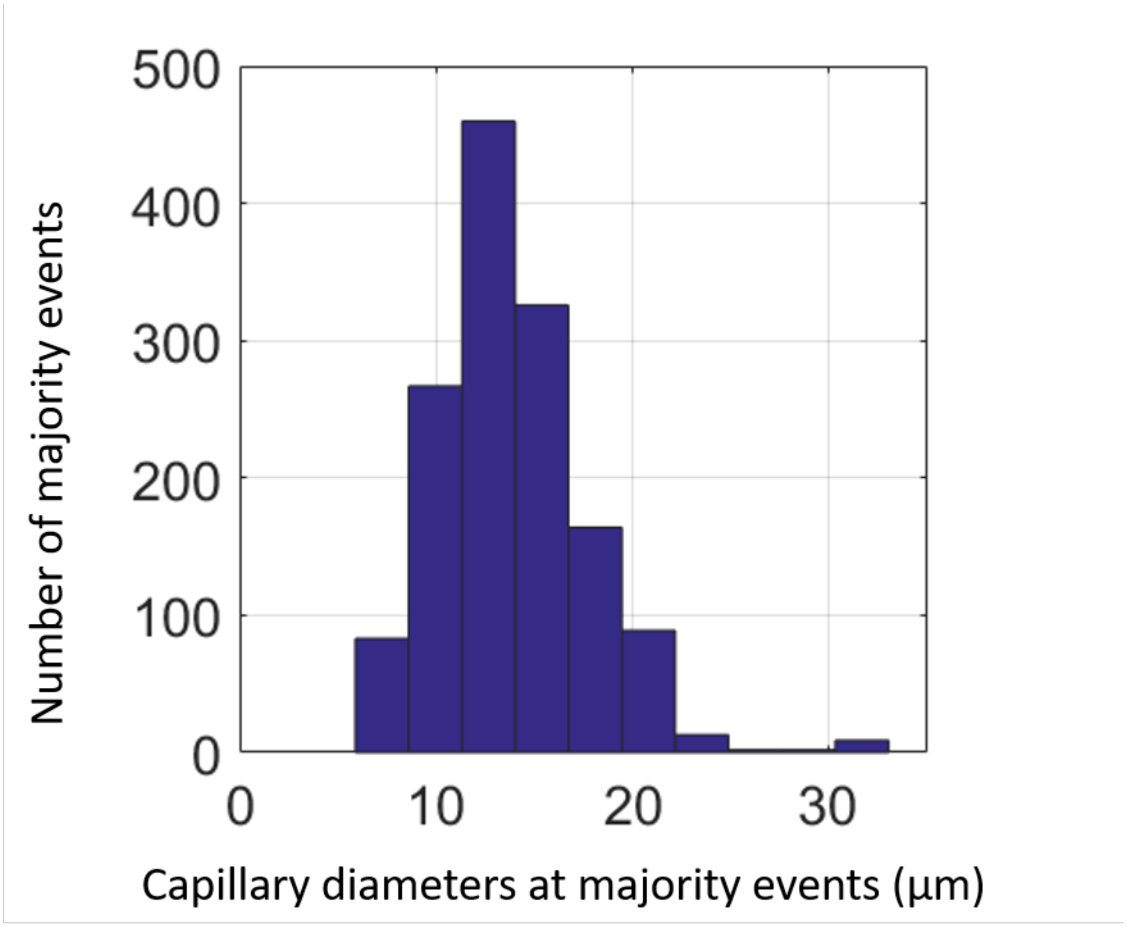

Fig. S19. Distribution of the capillary diameters at the positions where majority events were detected. Based on the capillary segmentations, capillary diameters were computed at the average positions of all events occuring in the 106 capillaries of our study, and the diameter distribution generated accordingly. Most majority events were detected in capillary segments of approximately the same diameter as WBCs, *i.e.*, in the 10-20 µm range, thus confirming previous observations in the literature[15,33] and the usability of events as proxies of WBCs.

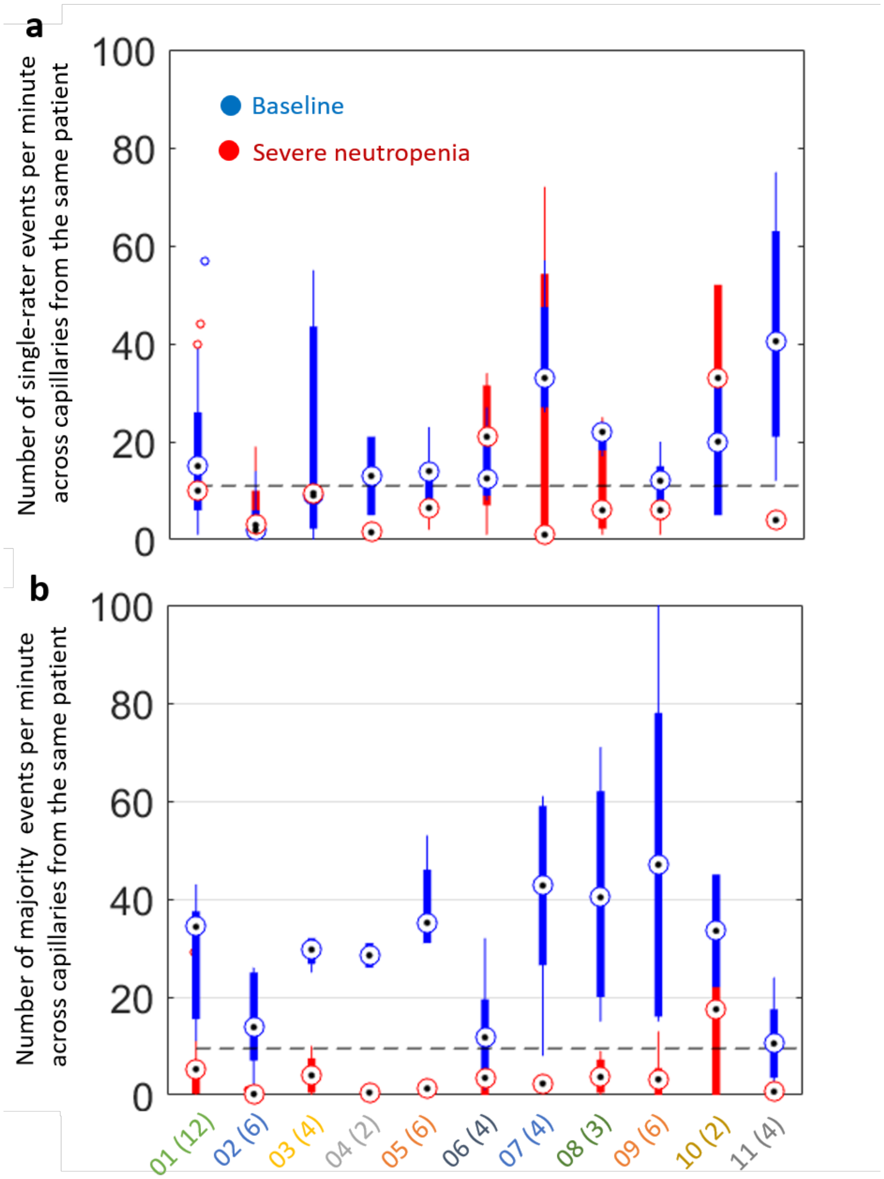

Fig. S20. Discrimination between baseline and severe neutropenia for single-rater versus majority events. (a) Counts of single-rater events. **(b)** Counts of majority-rater events. The median numbers of events observed per minute, when averaging all available capillaries per patient, are shown for baseline (blue dots) and severe neutropenia (red dots) for the 22 acquired video sets and 11 patients of our study. The corresponding cross-capillary variability is also shown for each patient (blue and red bars with notch extremes determined as $q_2 \pm 1.57(q_3 - q_1) / N^{1/2}$, where the q_i are the respective quartiles, and where N=11 is the amount of paired data points). For single-rater events, differences in patient counts between baseline and severe neutropenia did not reach statistical significance (Wilcoxon signed-rank test, N=11, P=0.17), with a median difference of only eight counts per minute. For majority events, differences in patient counts did reach statistical significance (Wilcoxon signed-rank test, N=11, P=0.00097), with a median difference of 25 counts per minute.The optimal thresholds to separate baseline from severe neutropenia are shown as dotted black lines. The X-axis is labeled with the patient IDs together with their amount of analyzed capillaries (brackets). The median amount of capillaries used per patient was four.

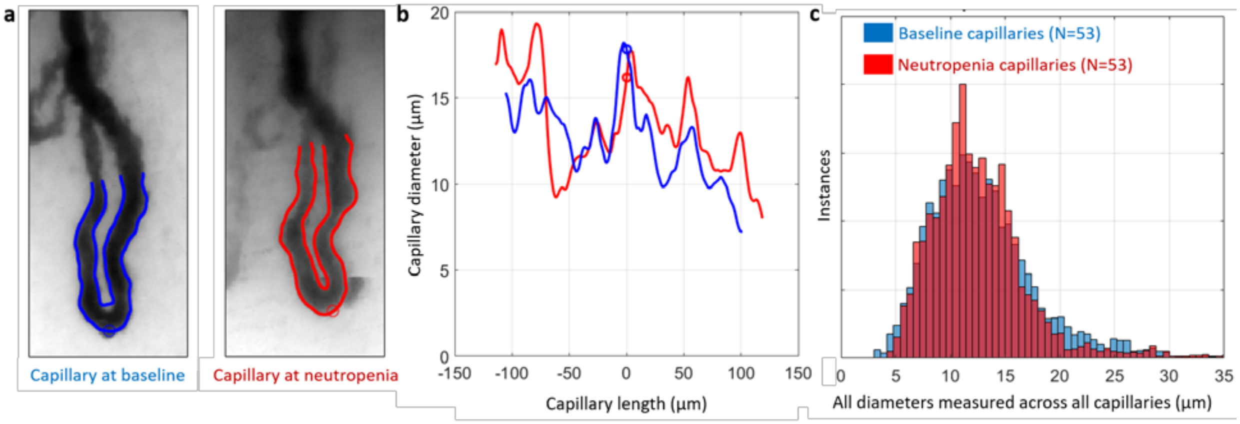

Fig. S21. Comparison of capillary diameter before versus after chemotherapy. (a) Segmentation of a single capillary outline before (blue) versus after (red) chemotherapy. The capillary tip–called *apex*–is labeled by a circle. **(b)** Diameter of that single capillary before (blue) and after (red) chemotherapy, taking the apex (blue and red circles respectively) as the reference point. **(c)** Histogram of all capillary diameters used in our study before (N=53, in blue) and after (N=53, in red) chemotherapy. For each capillary, the diameter was sampled in 1,000 locations along its length. The median diameter value was then determined for every capillary to generate the histograms before chemotherapy (baseline) and after chemotherapy (severe neutropenia). No statistically-significant variation in capillary diameter was observed between the two time points (Wilcoxon signed-rank test, *N*=53, *P*=0.504).

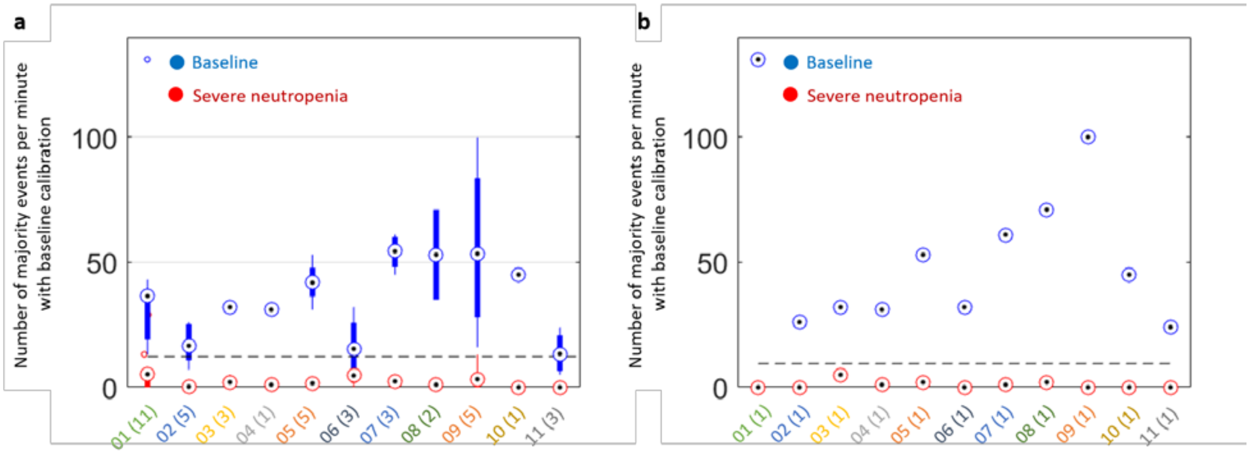

Fig. S22. Median number of majority events after performing a calibration at baseline. (a) For every patient, the capillary pair with least majority-event counts at baseline was removed. **(b)** For every patient, only the capillary pair with most majority-event counts at baseline was retained. In both cases, the separation between baseline (blue dots) and severe neutropenia (red dots) increased compared to the non-calibrated results (Fig. 7), and a perfect classification was achieved. The blue and red bars show the variability in the number of majority events for each patient across each individual capillary. The dotted black lines show the thresholds used to separate baseline from severe neutropenia. The X-axis is labeled with the patient IDs together with their number of available capillaries (in brackets).

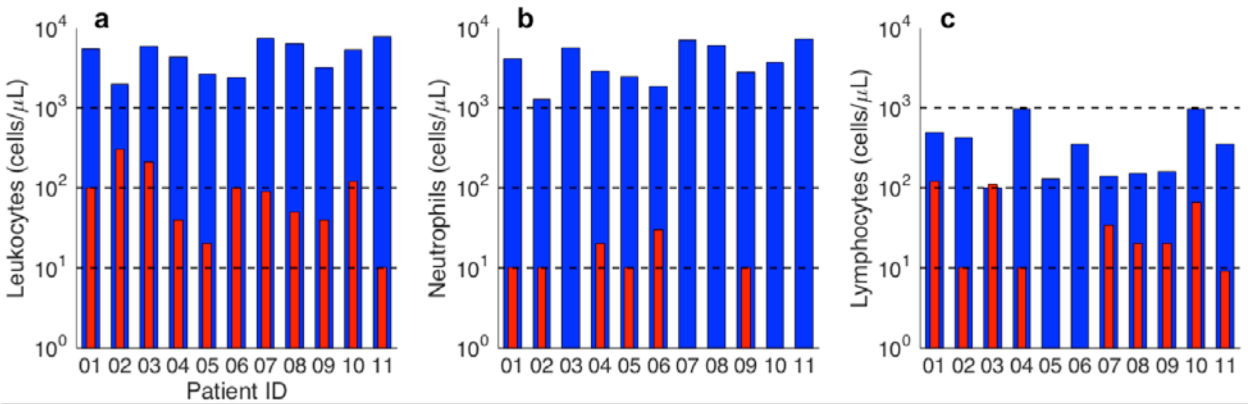

Fig. S23. Plots of reference WBC concentrations obtained from hospital clinical laboratory. (a) Leukocyte concentrations (logarithmic scale) for every patient at baseline (blue bars) versus severe neutropenia (red bars). **(b)** Idem for neutrophil concentrations **(c)** Idem for lymphocyte concentrations.

Supplementary movies

Movie S1. Example pair of raw videos acquired from a patient at baseline and severe neutropenia with our optical prototype.

Link to: https://www.dropbox.com/s/w52w905ssrlk83v/Movie%20S1.mp4?dl=0

Link to: https://youtu.be/q_3H6pKyhb8

Movie S2. Example capillary video illustrating the passage of one event in a capillary.

Link to: https://www.dropbox.com/s/shfxspnkzm8kuyp/Movie%20S2.avi?dl=0

Link to: https://youtu.be/LaTPRGgSWBg

Movie S3. Example full-minute capillary video with rater marks and ST map.

Link to: https://www.dropbox.com/s/spgqueuzzvwtdhz/Movie%20S3.mov?dl=0

Link to: https://youtu.be/Kddg2aGSlLg

Movie S4. Full-minute capillary video of Patient 06 with low baseline count.

Link to: https://www.dropbox.com/s/l0bhqdh7stgjjr0/Movie%20S4.mov?dl=0

Link to: https://youtu.be/zVSRje1QEWw

Supplementary references

[R1] Mattes, D., Haynor, D. R., Vesselle, H., Lewellyn, T. K. & Eubank, W. Nonrigid multimodality image registration. In Med. Imaging 2001, 1609–1620 (2001).

[R2] Lee, J. H. et al. Measurement of very low concentration of microparticles in fluid by single particle detection using acoustic radiation force induced particle motion. In 2015 IEEE Int. Ultrason. Symp., 1–4 (2015).

[R3] Tam, J., Tiruveedhula, P. & Roorda, A. Characterization of single-file flow through human retinal parafoveal capillaries using an adaptive optics scanning laser ophthalmoscope. Biomed. Opt. Express. 2, 781–793 (2011).

[R4] Allen, P. D., Taylor, C. J., Herrick, A. L. & Moore, T. Enhancement of temporally variable features in nailfold capillary patterns. In BMVC, 1–10 (1998).

[R5] Kass, M., Witkin, A. & Terzopoulos, D. Snakes: Active contour models. Int. J. Comput. Vision. 4, 321–331 (1988).
